# Racial inequalities in mental healthcare use and mortality: a cross-sectional analysis of 1.2 million low-income individuals in Rio de Janeiro, Brazil 2010–2016

**DOI:** 10.1136/bmjgh-2023-013327

**Published:** 2023-12-02

**Authors:** Sophia Medeiros, Rony Coelho, Christopher Millett, Valeria Saraceni, Claudia Medina Coeli, Anete Trajman, Davide Rasella, Betina Durovni, Thomas Hone

**Affiliations:** 1 Public Health Policy Evaluation Unit, School of Public Health, Imperial College London, London, UK; 2 Instituto de Estudos para Políticas de Saúde, São Paulo, Brazil; 3 NOVA National School of Public Health, Public Health Research Centre, Comprehensive Health Research Center, NOVA University Lisbon, Lisboa, Portugal; 4 Health Surveillance Branch, Secretaria Municipal de Saúde do Rio de Janeiro, Rio de Janeiro, Brazil; 5 Instituto de Estudos em Saúde Coletiva, Universidade Federal do Rio de Janeiro, Rio de Janeiro, Brazil; 6 Programa de Pós-graduação em Clínica Médica, Universidade Federal do Rio de Janeiro, Rio de Janeiro, Brazil; 7 Instituto de Saúde Coletiva, Universidade Federal da Bahia, Salvador, Brazil; 8 Centro de Estudos Estratégicos, Fundação Oswaldo Cruz, Rio de Janeiro, Brazil

**Keywords:** Mental Health & Psychiatry, Health systems

## Abstract

**Introduction:**

Mental health inequalities across racial and ethnic groups are large and unjust in many countries, yet these inequalities remain under-researched, particularly in low-income and middle-income countries such as Brazil. This study investigates racial and socioeconomic inequalities in primary healthcare usage, hospitalisation and mortality for mental health disorders in Rio de Janeiro, Brazil.

**Methods:**

A cohort of 1.2 million low-income adults from Rio de Janeiro, Brazil with linked socioeconomic, demographic, healthcare use and mortality records was cross-sectionally analysed. Poisson regression models were used to investigate associations between self-defined race/colour and primary healthcare (PHC) usage, hospitalisation and mortality due to mental disorders, adjusting for socioeconomic factors. Interactions between race/colour and socioeconomic characteristics (sex, education level, income) explored if black and pardo (mixed race) individuals faced compounded risk of adverse mental health outcomes.

**Results:**

There were 272 532 PHC consultations, 10 970 hospitalisations and 259 deaths due to mental disorders between 2010 and 2016. After adjusting for a wide range of socioeconomic factors, the lowest PHC usage rates were observed in black (adjusted rate ratio (ARR): 0.64; 95% CI 0.60 to 0.68; compared with white) and pardo individuals (ARR: 0.87; 95% CI 0.83 to 0.92). Black individuals were more likely to die from mental disorders (ARR: 1.68; 95% CI 1.19 to 2.37; compared with white), as were those with lower educational attainment and household income. In interaction models, being black or pardo conferred additional disadvantage across mental health outcomes. The highest educated black (ARR: 0.56; 95% CI 0.47 to 0.66) and pardo (ARR: 0.75; 95% CI 0.66 to 0.87) individuals had lower rates of PHC usage for mental disorders compared with the least educated white individuals. Black individuals were 3.7 times (ARR: 3.67; 95% CI 1.29 to 10.42) more likely to die from mental disorders compared with white individuals with the same education level.

**Conclusion:**

In low-income individuals in Rio de Janeiro, racial/colour inequalities in mental health outcomes were large and not fully explainable by socioeconomic status. Black and pardo Brazilians were consistently negatively affected, with lower PHC usage and worse mental health outcomes.

**WHAT IS ALREADY KNOWN ON THIS TOPIC**

- Race/ethnicity is an important social determinant of mental disorders and contributes to inequalities in mental healthcare use and mental health outcomes resulting from barriers to access, biases in diagnosis and treatment, and wider socioeconomic inequalities in education and employment.
- Evidence on racial/ethnic inequalities in mental health is predominantly from high-income countries, with high-quality evidence from low/middle-income countries lacking.
- Mental disorders are strongly associated with social inequalities including poverty, financial hardship and low educational attainment.
- The most common mental disorders including depression and anxiety are more prevalent in women, racial/ethnic minorities and individuals with disabilities.

**WHAT THIS STUDY ADDS**

- This study addresses multiple mental disorders frequently omitted in Brazilian research that typically focusses only on common mental disorders including anxiety and depression.
- This study assesses the interaction of multiple racial/colour and socioeconomic characteristics in mental healthcare and health outcomes in a large cohort of low-income Brazilian individuals, with linked socioeconomic, demographic, healthcare use and mortality records.
- Black and pardo Brazilians lying at the intersection of multiple axes of inequality disproportionately face poorer mental health outcomes compared with white Brazilians.
- This study highlights the socioeconomic gradient in mental healthcare among low-income individuals, quantifying the racial/colour inequalities that persist in already deprived groups.

**HOW THIS STUDY MIGHT AFFECT RESEARCH, PRACTICE OR POLICY**

- Racial and ethnic health inequalities remain a sizeable challenge for many countries.
- Understanding why people of non-white racial/colour groups, such as black and pardo Brazilians, experience lower rates of healthcare use and poorer health outcomes not fully explainable by socioeconomic status is vital for strengthening services.
- Policymakers should focus on equal distribution of services across the city and country’s regions to increase accessibility for all socioeconomic groups and minimise the existing treatment gap in mental healthcare.

## Introduction

Mental disorders are among the top 10 leading causes of the global disease burden.^1^ Poor mental health costs the global economy US$2.5 trillion annually and is expected to rise to US$6 trillion by 2030.^2^ The United Nations Sustainable Development Goals identified mental health as a priority for global development, and goal 3.4 aims to reduce premature mortality by one-third from non-communicable diseases, including mental health, by 2030.^3^ Although 80% of individuals with mental disorders reside in low/middle-income countries (LMICs),^1^ data on mental disorders in LMICs is limited due to under-reporting and under-diagnosis.^4^

Discrimination and racism has been associated with depression and anxiety, psychological distress, and psychiatric disorders in multiple settings.^5^ However, broader patterns and underlying mechanisms of racial inequalities in mental health outcomes in LMICs remain unclear.^6^ Socioeconomic factors may intersect with race/ethnicity to confer additional disadvantage in ways different to that seen in high-income settings, generating complex patterns of inequality.^7 8^ For example, individuals from certain racial and ethnic groups living in LMICs experience severe discrimination, poverty, unemployment, and lack of access to high-quality education, resources, and healthcare, and often reside in violent/high crime neighbourhoods.^7–10^ This, in turn, can contribute to especially poor mental health outcomes through financial stress and exposure to chronic and acute stressors.^8^

Brazil is an important setting to evaluate inequalities in access to mental healthcare services and outcomes between racial groups. Brazil was the last country in the Western world to abolish slavery and to this day, pervasive racial and socioeconomic inequality permeate Brazilian culture and society.^11^ For example, 9.1% of black and pardo (mixed race) Brazilians are illiterate compared with 3.9% of white Brazilians, and while 15.4% of white Brazilians earn less than US$5.50 a day, this number more than doubles for black and pardo Brazilians (32.9%).^12^ The city of Rio de Janeiro in Brazil is an important setting for this research. It is highly unequal; in 2010 its GINI index was 0.6392.^13^ An estimated one-fifth of Rio de Janeiro’s population lives in informal settlements known as *favelas*.^14 15^ In 2010, 51.2% of the 6.3 million inhabitants self-classified as white, 11.5% as black and 36.5% as pardo (mixed race).^13^ In 2017, 71.1% of the total population in Rio de Janeiro was covered by primary healthcare (PHC).^16^ Lack of infrastructure, inadequate housing, informal employment, violence, marginalisation of the poor, and insufficient public services drive social and health inequalities.

Few studies have explored associations between race and mental healthcare usage and mental health outcomes in Brazil, with previous work producing mixed findings.^17–21^ Evidence from the Brazilian National Health Survey suggests there is no association between poor mental health and skin colour in the country,^18^ yet a recent systematic review found a higher prevalence of mental disorders in non-white Brazilians.^17^ Furthermore, research frequently focuses on anxiety and depression, neglecting other mental disorders,^22–25^ and there have been few studies investigating racial/colour and socioeconomic inequalities beyond prevalence.^26–33^ The intersection of multiple socioeconomic characteristics with race in mental healthcare and health outcomes have not been previously explored in Brazil.

This study investigates racial/colour patterns of PHC usage, hospitalisation and mortality for mental health disorders in a cohort of 1.2 million low-income individuals in Rio de Janeiro, Brazil. This study aims to fill an important knowledge gap using individual-level data with linked healthcare usage data with a wide range of socioecononomic variables, considering both primary and secondary causes of healthcare usage. With robust quantification of racial/colour inequalities, this study explores to what extent these inequalities persist after adjusting for socioeconomic factors and also explores how these patterns vary with multiple socioeconomic disadvantage, particularly for black and pardo Brazilians. In reference to racial inequalities, Brazilian literature primarily and formally uses the term ‘race/colour’ (with the term ‘ethnicity’ reserved to distinguish indigenous peoples in Brazil), therefore, this paper will use ‘race/colour’ in relation to Brazil and ‘race/ethnicity’ when referring to international literature.^34^

## Methods

This study is a cross-sectional analysis of a cohort of 1.2 million low-income individuals with linked welfare, PHC, hospitalisation and mortality records.

### Data sources

The study population was composed of adults (aged 15+ years) registered in the *Cadastro Único* database between 1 January 2010 and 31 December 2014. The *Cadastro Único* is a national administrative database of all individuals claiming government welfare and covers approximately 25% of the city’s (Rio de Janeiro) population. As the welfare programmes are targeted towards low-income families, the study population is generally lower-income and lives in poorer neighbourhoods of the city. *Cadastro Único* records were linked to three additional datasets: PHC electronic health records (from *Estratégia Saúde da Família* (Family Health Strategy); ESF), public hospitalisation records (*Sistema de Informações Hospitalares*); mortality records (*Sistema de Informações sobre Mortalidade*), all covering the period from 1 January 2010 to 31 December 2016. The datasets were linked via a combination of deterministic and probabilistic approaches, with details published elsewhere (online supplemental material 1).^35^ From PHC consultations, hospital admission and mortality records, International Classification of Disease (ICD-10) codes and International Classification of Primary Care (ICPC) codes were obtained for any mental disorder.

10.1136/bmjgh-2023-013327.supp1 Supplementary data

### Outcomes

Three outcomes were considered for mental health: PHC usage (number of consultations as part of ESF), hospitalisation (count) and mortality for any mental disorder. Both primary and secondary causes of PHC usage were used to determine if consultations included mental health conditions. Mental disorders were grouped into six main categories with associated ICD-10 and ICPC codes (online supplemental material 2) to cover all relevant conditions^36–38^: substance abuse/dependence disorders (ICD-10 F10–F19; ICPC P15, P17–P19); psychotic syndromes (ICD-10 F20–F25, F28, F29; ICPC P29, P71–P73, P98, P99); mood affective disorders (ICD-10 F30–F34, F38, F39; ICPC P03, P76); neurotic, stress-related and somatoform disorders (hereafter referred to as anxiety disorders) (ICD-10 F40–F45, F48; ICPC P01, P02, P74, P79, P82); personality and behaviour disorders (ICD-10 F50, F51, F60–F63, F91–F94; ICPC P80, P86); suicide/associated outcomes (ICD-10 X60-X84, R45.851; ICPC P77). All relevant ICD-10 and ICPC codes for mental disorders were analysed by the six main categories and also in total (ie, any mental health conditions from any category). This was to record the maximum number of contacts with the health system and to account for comorbidity of psychiatric disorders.^39^

10.1136/bmjgh-2023-013327.supp2 Supplementary data

### Variables

The main variable of interest was race (based on self-reported skin colour in Brazil—black, white, pardo (mixed race), other) obtained from the *Cadastro Único*. Other key variables were sex (male, female), highest educational attainment (none/preschool/literacy class, elementary school, high school or higher education) and household per capita income decile (Q1–Q10; for low-income individuals).

A range of other individual-level and household-level sociodemographic categorical variables were included as potential confounders associated with both race/colour and mental health outcomes.^8 40^ Individual-level covariates included: age group in years (15–19, 20–22, 23–24, 25–29, 30–34, 35–39, 40–44, 45–49, 50–59, 60–69, 70 or more); disability (yes, no) and employment status (unemployed—yes, no). Household-level covariates included whether the family receives *Bolsa Família*—a conditional cash transfer programme for families earning below US$70 per day (yes, no)^41^; number of family members per bedroom (two or fewer, more than two, three or fewer, more than three, four or fewer, more than four); household flooring (soil, cement, repurposed wood, ceramics/tiles, other); household piped water access (yes, no); formal employment in the family (yes, no); quintiles of per capita expenditure on medicines (Q1–Q5); quintiles of per capita expenditure on food (Q1–Q5).

Two variables related to employment were included. The first variable—formal employment in the family—reflects favourable job market conditions such as higher income, access to health insurance and job security, potentially impacting household welfare and the mental health of all residents. The second variable—individual employment status—was included to capture the well-established relationship between employment and mental health outcomes. Both variables were included to address scenarios where variations in employment types within households may lead to different risks of mental health outcomes.^7 10 40^

### Statistical analyses

Individuals entered the cohort between 1 January 2010 and 31 December 2014 (when they joined *Cadastro Único*). An individual’s end of observation period was either their date of death or 31 December 2016. The analysis was restricted to individuals who had turned 15 years by 31 December 2016. Amarelo (Asian) and indigenous individuals were aggregated as ‘other’ race/colour due to low numbers. The total numbers of mental disorder associated PHC consultations, hospitalisations and deaths were reported, overall and by mental disorder classification. Crude rates were estimated (per 100 000 person-years of observation) overall and by socioeconomic variables.

First, separate multivariable Poisson regression models were employed to explore the association between race/colour and the three outcomes. All individuals in the cohort were included in the analysis for hospitalisation and mortality outcomes, regardless of prior mental disorder history. For PHC usage, only ESF registered individuals were included (as not all individuals in *Cadastro Único* were registered to use the ESF), also regardless of prior mental disorder history. All socioeconomic covariates specified above were included in each model, with full regression results reported. A Poisson specification was deemed appropriate to model both count and binary data with an observation time offset (ie, allowing a variable observation time per individual).^42 43^ This resulted in modelled outcomes expressed per 100 000 person-years. Coefficients were exponentiated and reported as adjusted rate ratios (ARRs). As all explanatory socioeconomic variables were expressed categorically, these were interpreted as the ratio of the category of interest (eg, female) and the baseline category (eg, male). An ARR <1 indicated that the modelled rate was lower in the category of interest compared with the baseline category, while an ARR >1 indicated a higher rate. Covariates were tested for collinearity and all variance inflation factors were less than two. Robust standard errors (SEs) were used to control for mild violation of underlying assumptions.^44^ For hospitalisation and mortality, additional analyses were conducted by repeating the above models and additionally adjusting for PHC usage. This tested whether racial/colour inequalities persisted after adjusting for socioeconomic factors and also PHC usage.

Second, the models were expanded with two-way interactions between race/colour and the variables sex, education level and deciles of income as an intersectionality analysis. These explored associations between different racial/colour-sociodemographic stratifications and the outcomes, given known interconnected relationships and potential combined contributions to health inequalities.^24 45 46^ Interactions were first tested for statistical significance and, if statistically significant, postregression predicted rates were plotted. Poisson regression models for all interactions were adjusted for all socioeconomic variables.

ARRs with 95% CIs and p values were reported. All analyses used robust SEs and were performed in Stata V.15.1 (StataCorp; College Station, Texas, USA).

## Results

A total of 1 243 932 adults (representing 7 415 250 person-years) aged 15+ years were included in the analysis (table 1). The mean observation period was 5.96 years. Over half the population was female (753 601; 60.6%). By race/colour, 50.7% self-identified as pardo, 29.6% as white and 17.5% as black. Most (1 140 135; 91.7%) had elementary, or high school or higher education, while 8.3% (103 797) had no formal education beyond preschool. There was a total of 272 532 mental disorder-associated PHC consultations, 10 970 hospitalisations and 259 deaths in the period. The annual rate of mental disorder-associated PHC consultations, hospitalisations and mortality was 446.11, 21.10 and 0.50 per 100 000 person-years, respectively.

**Table 1:** Characteristics of the study population

Characteristics	Entire cohort		ESF registered	
	N	Proportion (%)	N	Proportion (%)
Individual				
Sex				
Male	490 331	39.4	264 883	35.6
Female	753 601	60.6	478 863	64.4
Race/colour				
White	368 037	29.6	217 573	29.3
Black	217 089	17.5	131 156	17.6
Pardo (mixed)	630 787	50.7	379 861	51.1
Other	28 019	2.3	15 156	2.0
Education level				
None/preschool/literacy class	103 797	8.3	55 341	7.4
Elementary	759 412	61.1	457 203	61.5
High school or higher education	380 723	30.6	231 202	31.1
Age group (years)				
15–19	218 258	17.6	131 709	17.7
20–22	118 964	9.6	68 964	9.3
23–24	70 921	5.7	40 074	5.4
25–29	135 023	10.9	77 041	10.4
30–34	113 225	9.1	67 970	9.1
35–39	115 473	9.3	70 191	9.4
40–44	106 051	8.5	64 690	8.7
45–49	92 834	7.5	56 895	7.7
50–59	143 465	11.5	89 167	12.0
60–69	82 633	6.6	51 668	7.0
70+	47 085	3.8	25 377	3.4
Disability				
No	1 196 694	96.2	713 398	95.9
Yes	47 238	3.8	30 348	4.1
Unemployed				
No	917 158	73.7	533 677	71.8
Yes	326 774	26.3	210 069	28.2
Household				
Deciles of income				
Q1 (poorest)	119 034	9.6	63 341	8.5
Q2	110 703	8.9	67 373	9.1
Q3	114 473	9.2	70 686	9.5
Q4	117 367	9.4	71 607	9.6
Q5	122 216	9.8	74 144	10.0
Q6	121 919	9.8	73 753	9.9
Q7	123 778	10.0	75 807	10.2
Q8	127 685	10.3	78 796	10.6
Q9	133 704	10.8	80 641	10.8
Q10 (richest)	153 053	12.3	87 598	11.8
*Bolsa Família*-claiming family				
No	415 885	33.4	222 494	29.9
Yes	828 047	66.6	521 252	70.1
Family members per bedroom				
2 or fewer	498 746	40.1	305 150	41.0
More than 2, 3 or fewer	312 434	25.1	189 599	25.5
More than 3, 4 or fewer	223 231	18.0	131 366	17.7
More than 4	209 521	16.8	117 631	15.8
Household flooring material				
Soil	297 176	23.9	151 942	20.4
Cement	228 487	18.4	145 036	19.5
Repurposed wood	23 817	1.9	13 377	1.8
Ceramics/tiles	672 504	54.1	421 054	56.6
Other	21 948	1.8	12 337	1.7
Piped water access				
No	34 746	2.8	17 940	2.4
Yes	1 209 186	97.2	725 806	97.6
Formal employment in the family				
No	976 946	78.5	579 277	77.9
Yes	266 986	21.5	164 469	22.1
Quintiles of per capita expenditure on medicines				
Q1 (least)	995 779	80.1	590 793	79.4
Q2	90 943	7.3	58 729	7.9
Q3	65 390	5.3	40 106	5.4
Q4	47 528	3.8	28 879	3.9
Q5 (most)	44 292	3.6	25 239	3.4
Quintiles of per capita expenditure on food				
Q1 (least)	273 322	22.0	148 553	20.0
Q2	240 222	19.3	148 933	20.0
Q3	250 127	20.1	153 508	20.6
Q4	247 494	19.9	150 499	20.2
Q5 (most)	232 767	18.7	142 253	19.1
Total individuals	1 243 932	100	743 746	100

ESF, Estratégia Saúde da Família (Family Health Strategy).

**Figure 1:**
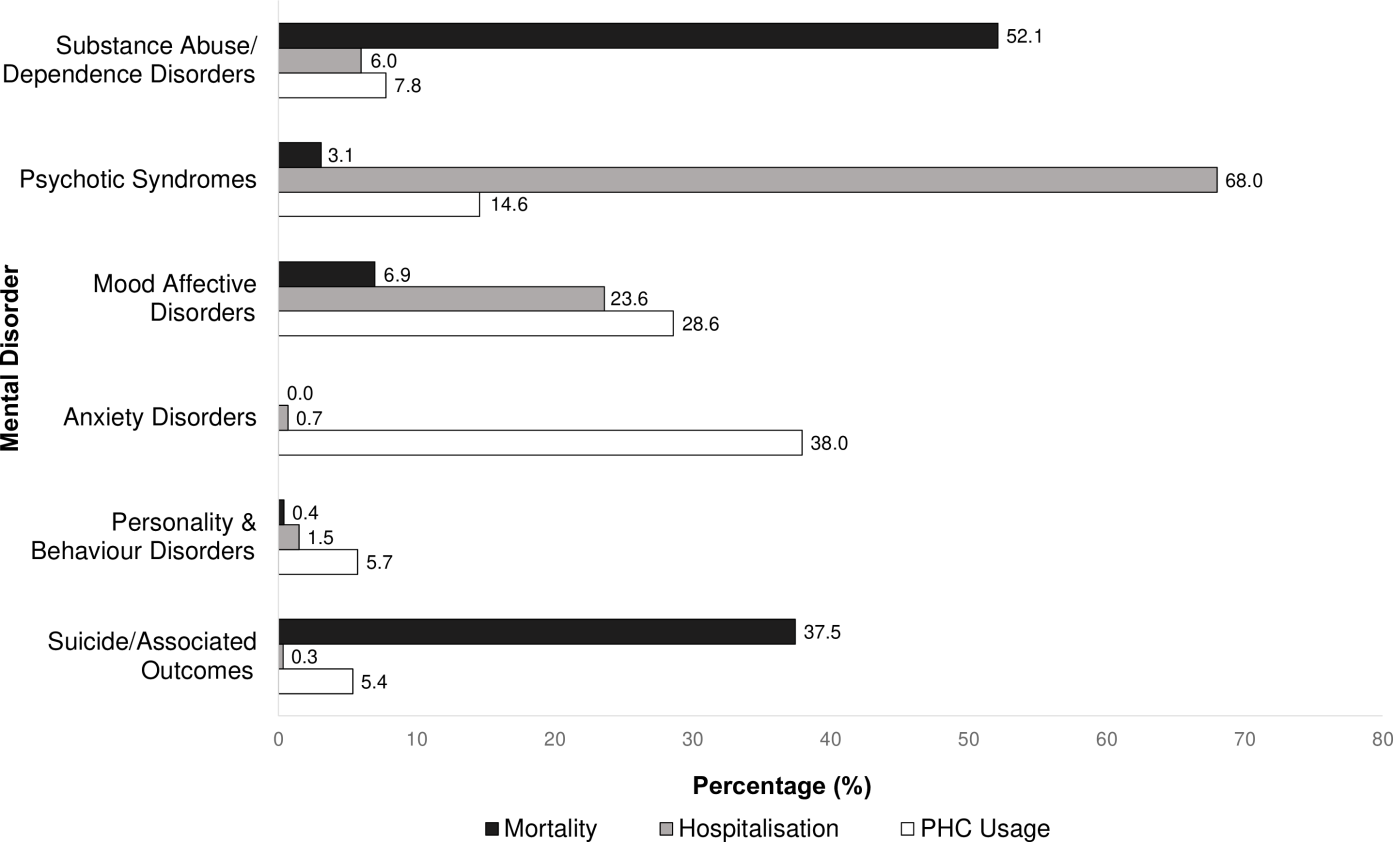
Proportion of mental disorder associated PHC utilisation (consultations), hospitalisations, and mortality by mental disorder classification between 2010-2016 in a cohort of low-income individuals in Rio de Janeiro, Brazil.PHC, primary healthcare.

The burden of different mental disorders varied considerably across PHC usage, hospitalisations and mortality (figure 1). For PHC usage, anxiety disorders comprised the greatest share of PHC consultations (38.0%), followed by mood affective disorders (29.0%). Psychotic syndromes (68.0%) comprised over two-thirds of mental disorder hospitalisations, followed by mood affective disorders (23.6%). Nearly 90% of all mental disorder-related deaths were attributable to substance abuse/dependence disorders (52.1%) or suicide/associated outcomes (37.5%).

Annual rates of mental disorder associated PHC usage, hospitalisation and mortality per 100 000 person-years were calculated (table 2; online supplemental material 3 for all covariates). Rates of PHC usage were lowest in black (593.7 per 100 000 person-years), followed by pardo (697.5), then white (896.3) individuals, whereas hospitalisation rates were lowest in pardo (18.5), black (23.5), then white individuals (24.4). Mortality rates by racial/colour group revealed a higher mortality rate in individuals identifying as black (0.7) compared with pardo (0.5) and white (0.4) individuals.

10.1136/bmjgh-2023-013327.supp3 Supplementary data

**Table 2:** Annual rates of mental disorder associated PHC consultations, hospitalisations and mortality by all sociodemographic variables per 100 000 person-years*

Characteristics	PHC usage				Hospitalisation			Mortality		
	N	Proportion (%)	Rate (per 100 000 person-years)		N	Proportion (%)	Rate (per 100 000 person-years)	N	Proportion (%)	Rate (per 100 000 person-years)
			Entire cohort	ESF registered						
Individual										
Sex										
Male	6615.6	2.9	224.6	412.8	801.4	7.3	27.2	23.3	9.0	0.8
Female	26 464.9	11.4	592.1	923.7	762.9	7.0	17.1	13.7	5.3	0.3
Race/colour										
White	11 599.9	5.0	534.8	896.3	529.1	4.8	24.4	8.7	3.4	0.4
Black	4768.1	2.1	363.0	593.7	308.3	2.8	23.5	9.4	3.6	0.7
Pardo (mixed)	16 006.3	6.9	421.9	697.5	702.7	6.4	18.5	18.3	7.1	0.5
Other	706.1	0.3	509.0	934.8	24.1	0.2	17.4	0.6	0.2	0.4
Education level										
None/preschool/literacy class	3075.4	1.3	474.4	877.8	194.1	1.8	29.9	5.0	1.9	0.8
Elementary	21 112.3	9.1	457.4	754.5	946.3	8.6	20.5	25.9	10.0	0.6
High school or higher education	8892.7	3.8	413.4	674.1	423.9	3.9	19.7	6.1	2.4	0.3
Household										
Deciles of income										
Q1 (poorest)	2702.7	1.2	369.7	689.8	195.7	1.8	26.8	7.1	2.8	1.0
Q2	2811.0	1.2	404.3	662.3	117.1	1.1	16.8	4.3	1.7	0.6
Q3	2867.3	1.2	400.3	645.2	120.7	1.1	16.9	3.9	1.5	0.5
Q4	3018.7	1.3	410.7	670.8	98.4	0.9	13.4	3.1	1.2	0.4
Q5	2746.7	1.2	356.0	585.1	85.9	0.8	11.1	3.0	1.2	0.4
Q6	2603.4	1.1	341.7	562.9	92.1	0.8	12.1	2.3	0.9	0.3
Q7	2962.7	1.3	385.6	626.0	123.1	1.1	16.0	2.3	0.9	0.3
Q8	3174.9	1.4	409.8	658.9	145.1	1.3	18.7	3.3	1.3	0.4
Q9	3974.0	1.7	530.3	868.7	173.4	1.6	23.1	2.3	0.9	0.3
Q10 (richest)	6219.0	2.7	874.1	1508.0	412.6	3.8	58.0	5.4	2.1	0.8

*For main variables of interest. See online supplemental material 3 for all covariates.

ESF, Estratégia Saúde da Família (Family Health Strategy); PHC, primary healthcare.

PHC usage rates for mental disorders were lower in males than females, but hospitalisations and mortality were both greater for males. Rates for all outcomes decreased with greater educational attainment (table 2). General trends showed that individuals of higher-income households (noting that the overall cohort is generally lower-income) reported greater rates of PHC usage, with substantially higher rates for those in the highest-income decile. Increasing age was associated with higher rates of PHC usage and mortality. Those with a disability, who were unemployed, not receiving *Bolsa Família* or having higher household expenditures on medicine had higher levels of PHC usage, hospitalisation and mortality.

In adjusted regression models (table 3), black and pardo individuals had, respectively, a 36% (ARR: 0.64; 95% CI 0.60 to 0.68; p<0.001) and 13% (ARR: 0.87; 95% CI 0.83 to 0.92; p<0.001) lower rate of PHC usage for mental disorders compared with white individuals. There were also differences in hospitalisations. Pardo individuals had a 17% (ARR: 0.83; 95% CI 0.70 to 0.99; p<0.05) lower rate of hospitalisations compared with white individuals. Black individuals had a 7% (ARR: 0.93; 95% CI 0.75 to 1.15; p>0.05) lower rate of hospitalisations compared with white individuals, though this was not statistically significant. Black individuals were 1.7 times (ARR: 1.68; 95% CI 1.19 to 2.37; p<0.01) more likely to die from mental disorders compared with white individuals. In supplementary models, additionally adjusting for PHC usage in hospitalisation and mortality outcomes did not change the coefficients and estimates of inequality by racial/colour groups (online supplemental material 4).

10.1136/bmjgh-2023-013327.supp4 Supplementary data

**Table 3:** Adjusted rates ratios from Poisson regression models of mental disorder associated PHC consultations, hospitalisations and mortality by all sociodemographic variables

Characteristics	PHC usage		Hospitalisation		Mortality	
	ARR	95% CI	ARR	95% CI	ARR	95% CI
Individual						
Sex						
Male	1 (ref)	–	1 (ref)	–	1 (ref)	–
Female	1.79***	(1.69 to 1.89)	0.57***	(0.49 to 0.67)	0.32***	(0.25 to 0.42)
Race/colour						
White	1 (ref)	–	1 (ref)	–	1 (ref)	–
Black	0.64***	(0.60 to 0.68)	0.93	(0.75 to 1.15)	1.68**	(1.19 to 2.37)
Pardo (mixed)	0.87***	(0.83 to 0.92)	0.83*	(0.70 to 0.99)	1.27	(0.94 to 1.73)
Other	0.86*	(0.74 to 1.00)	0.55**	(0.37 to 0.83)	0.78	(0.29 to 2.19)
Education level						
None/preschool/literacy class	1 (ref)	–	1 (ref)	–	1 (ref)	–
Elementary	0.89*	(0.81 to 0.97)	0.92	(0.70 to 1.22)	0.86	(0.60 to 1.24)
High school or higher education	0.83***	(0.76 to 0.92)	0.78	(0.57 to 1.07)	0.53**	(0.34 to 0.85)
Age group (years)						
15–19	1 (ref)	–	1 (ref)	–	1 (ref)	–
20–22	1.53***	(1.32 to 1.77)	3.09***	(2.14 to 4.46)	3.63**	(1.52 to 8.66)
23–24	2.10***	(1.76 to 2.51)	4.71***	(3.18 to 6.97)	6.46***	(2.71 to 15.43)
25–29	2.97***	(2.63 to 3.37)	6.19***	(4.29 to 8.93)	4.97***	(2.14 to 11.52)
30–34	5.02***	(4.47 to 5.65)	8.94***	(6.02 to 13.29)	5.69***	(2.36 to 13.67)
35–39	6.85***	(6.15 to 7.64)	8.98***	(6.21 to 13.00)	5.48***	(2.27 to 13.20)
40–44	8.40***	(7.55 to 9.36)	9.28***	(6.16 to 13.97)	11.03***	(4.96 to 24.54)
45–49	9.94***	(8.91 to 11.09)	6.34***	(4.32 to 9.29)	9.07***	(3.98 to 20.68)
50–59	11.07***	(10.00 to 12.26)	5.83***	(3.95 to 8.59)	13.32***	(6.15 to 28.85)
60–69	10.01***	(8.90 to 11.26)	2.27***	(1.43 to 3.58)	17.96***	(8.06 to 40.06)
70+	5.69***	(4.91 to 6.60)	1.42	(0.70 to 2.89)	19.56***	(7.96 to 48.06)
Disability						
No	1 (ref)	–	1 (ref)	–	1 (ref)	–
Yes	2.96***	(2.76 to 3.18)	13.71***	(11.42 to 16.46)	1.73*	(1.05 to 2.86)
Unemployed						
No	1 (ref)	–	1 (ref)	–	1 (ref)	–
Yes	1.61***	(1.53 to 1.69)	2.11***	(1.78 to 2.49)	1.19	(0.86 to 1.66)
Household						
Deciles of income						
Q1 (poorest)	1 (ref)	–	1 (ref)	–	1 (ref)	–
Q2	0.96	(0.86 to 1.06)	0.79	(0.57 to 1.08)	0.77	(0.49 to 1.22)
Q3	0.91	(0.82 to 1.01)	0.82	(0.60 to 1.11)	0.67	(0.42 to 1.08)
Q4	0.98	(0.88 to 1.09)	0.72*	(0.53 to 0.99)	0.56*	(0.34 to 0.92)
Q5	0.90*	(0.81 to 1.00)	0.65**	(0.48 to 0.89)	0.52*	(0.31 to 0.87)
Q6	0.85**	(0.76 to 0.94)	0.72	(0.50 to 1.04)	0.40**	(0.22 to 0.72)
Q7	0.91	(0.82 to 1.01)	0.96	(0.65 to 1.41)	0.40**	(0.22 to 0.71)
Q8	0.88*	(0.79 to 0.98)	1.02	(0.71 to 1.46)	0.55*	(0.33 to 0.92)
Q9	0.94	(0.85 to 1.04)	1.03	(0.73 to 1.45)	0.35**	(0.19 to 0.64)
Q10 (richest)	1.08	(0.96 to 1.21)	1.84**	(1.25 to 2.70)	0.6	(0.35 to 1.01)
Household						
*Bolsa Família*-claiming family						
No	1 (ref)	–	1 (ref)	–	1 (ref)	–
Yes	1.01	(0.95 to 1.07)	1.22*	(1.00 to 1.49)	1.18	(0.87 to 1.60)
Family members per bedroom						
2 or fewer	1 (ref)	–	1 (ref)	–	1 (ref)	–
More than 2, 3 or fewer	0.85***	(0.80 to 0.90)	0.68***	(0.55 to 0.83)	1.16	(0.84 to 1.61)
More than 3, 4 or fewer	0.76***	(0.70 to 0.81)	0.61***	(0.48 to 0.79)	0.94	(0.64 to 1.39)
More than 4	0.82***	(0.76 to 0.90)	0.67*	(0.49 to 0.93)	1	(0.68 to 1.48)
Household flooring material						
Soil	1 (ref)	–	1 (ref)	–	1 (ref)	–
Cement	1.19***	(1.09 to 1.28)	0.51***	(0.40 to 0.65)	1	(0.66 to 1.52)
Repurposed wood	1.1	(0.95 to 1.27)	0.76	(0.49 to 1.17)	1.11	(0.43 to 2.85)
Ceramics/tiles	1.13**	(1.05 to 1.22)	0.49***	(0.39 to 0.62)	0.96	(0.65 to 1.41)
Other	1.06	(0.90 to 1.25)	1.22	(0.79 to 1.86)	2.05*	(1.03 to 4.05)
Piped water access						
No	1 (ref)	–	1 (ref)	–	1 (ref)	–
Yes	0.72***	(0.61 to 0.84)	0.73	(0.48 to 1.12)	0.63	(0.37 to 1.09)
Formal employment in the family						
No	1 (ref)	–	1 (ref)	–	1 (ref)	–
Yes	0.92**	(0.86 to 0.98)	0.76*	(0.59 to 0.96)	0.98	(0.65 to 1.46)
Quintiles of per capita expenditure on medicines						
Q1 (least)	1 (ref)	–	1 (ref)	–	1 (ref)	–
Q2	1.12**	(1.04 to 1.20)	1.07	(0.77 to 1.48)	1.27	(0.81 to 2.00)
Q3	1.20***	(1.11 to 1.31)	0.88	(0.66 to 1.18)	0.88	(0.48 to 1.63)
Q4	1.30***	(1.18 to 1.44)	0.76	(0.54 to 1.07)	1.4	(0.77 to 2.54)
Q5 (most)	1.43***	(1.28 to 1.59)	0.71	(0.49 to 1.04)	1.34	(0.70 to 2.57)
Quintiles of per capita expenditure on food						
Q1 (least)	1 (ref)	–	1 (ref)	–	1 (ref)	–
Q2	0.80***	(0.75 to 0.86)	0.57***	(0.46 to 0.71)	0.66*	(0.46 to 0.95)
Q3	0.80***	(0.74 to 0.85)	0.62***	(0.50 to 0.78)	0.62*	(0.42 to 0.92)
Q4	0.80***	(0.74 to 0.86)	0.58***	(0.45 to 0.75)	0.58*	(0.38 to 0.88)
Q5 (most)	0.80***	(0.74 to 0.87)	0.53***	(0.40 to 0.70)	0.56*	(0.36 to 0.88)
Total observations (N)	1 243 932		1 243 932		1 243 932	

Separate fully adjusted Poisson regressions per outcome (PHC usage (ESF registered users only), hospitalisation and mortality); adjusted for sex, race/colour, education level, age group, disability, unemployment, household per capita income decile, number of family members per bedroom, household flooring, household piped water access, formal employment in the family, Bolsa Família-receiving family, quintiles of household expenditure on medicines and food.

Robust SEs. *p<0.05; **p<0.01; *** p<0.001.

ARR, adjusted rate ratios; PHC, primary healthcare.

Other socioeconomic inequalities persisted in adjusted regression models. Females had almost two times (ARR: 1.79; 95% CI 1.69 to 1.89; p<0.001) the rate of PHC usage compared with males. In contrast, females had a 43% (ARR: 0.57; 95% CI 0.49 to 0.67; p<0.001) lower rate of hospitalisation and were 68% (ARR: 0.32; 95% CI 0.25 to 0.42; p<0.001) less likely to die from mental disorders compared with males. Individuals with high school or higher education were almost 50% (ARR: 0.53; 95% CI 0.34 to 0.85; p<0.01) less likely to die from mental health disorders compared with those with no/preschool/literacy class education. Higher-income individuals (Q10) were 84% more likely (ARR: 1.84; 95% CI 1.25 to 2.70; p<0.01) to be hospitalised compared with their lower-income counterparts (Q1), contrasting with results for unemployment. Unemployed individuals had 1.6 times (ARR: 1.61; 95% CI 1.53 to 1.69; p<0.001) as many PHC consultations than those employed and were two times (ARR: 2.11; 95% CI 1.78 to 2.49; p<0.001) as likely to be hospitalised.

**Figure 2:**
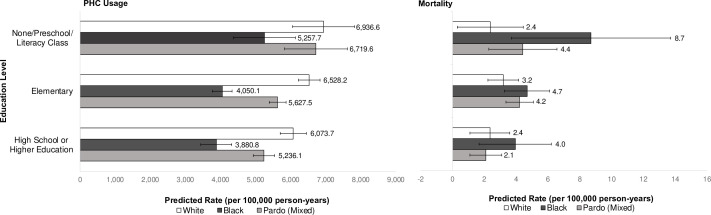
Predicted primary healthcare utilisation and mortality rates per 100,000 person-years based on adjusted Poisson regression models for interactions between race/ethnicity and education level with 95% confidence intervals displayed.Obtained from separate fully adjusted Poisson regressions per outcome (PHC utilisation [registered users only], hospitalisation, and mortality); adjusted for sex, age group, disability, unemployment, household per capita income decile, number of family members per bedroom, household flooring, household piped water access, formal employment in the family, Bolsa Família-receiving family, quintiles of household expenditure on medicines and food.

Two-way interactions between race/colour and other socioeconomic variables (sex, education level, deciles of income) were tested to explore intersectionality in the associations between mental health outcomes and socioeconomic factors. There were significant interactions between race/colour and education level for mental disorder associated PHC usage and mortality outcomes, but not hospitalisations (figure 2; online supplemental material 5). There was a general trend where black and pardo individuals had consistently lower rates of PHC usage (compared with white individuals) for all levels of education. For example, black and pardo individuals with high school or higher education had a 44% (ARR: 0.56; 95% CI 0.47 to 0.66; p<0.001) and 25% (ARR: 0.75; 95% CI 0.66 to 0.87; p<0.001) lower rate of PHC usage compared with the least educated white individuals, respectively. For mortality, black individuals had the highest mortality rates across all levels of education, but relative and absolute inequalities were largest for those with the lowest education. Thus, black and low education individuals were at increased risk of mortality for mental disorders (than the separate effects of being black and having low educational attainment on their own) (online supplemental material 6).

10.1136/bmjgh-2023-013327.supp5 Supplementary data

10.1136/bmjgh-2023-013327.supp6 Supplementary data

**Figure 3:**
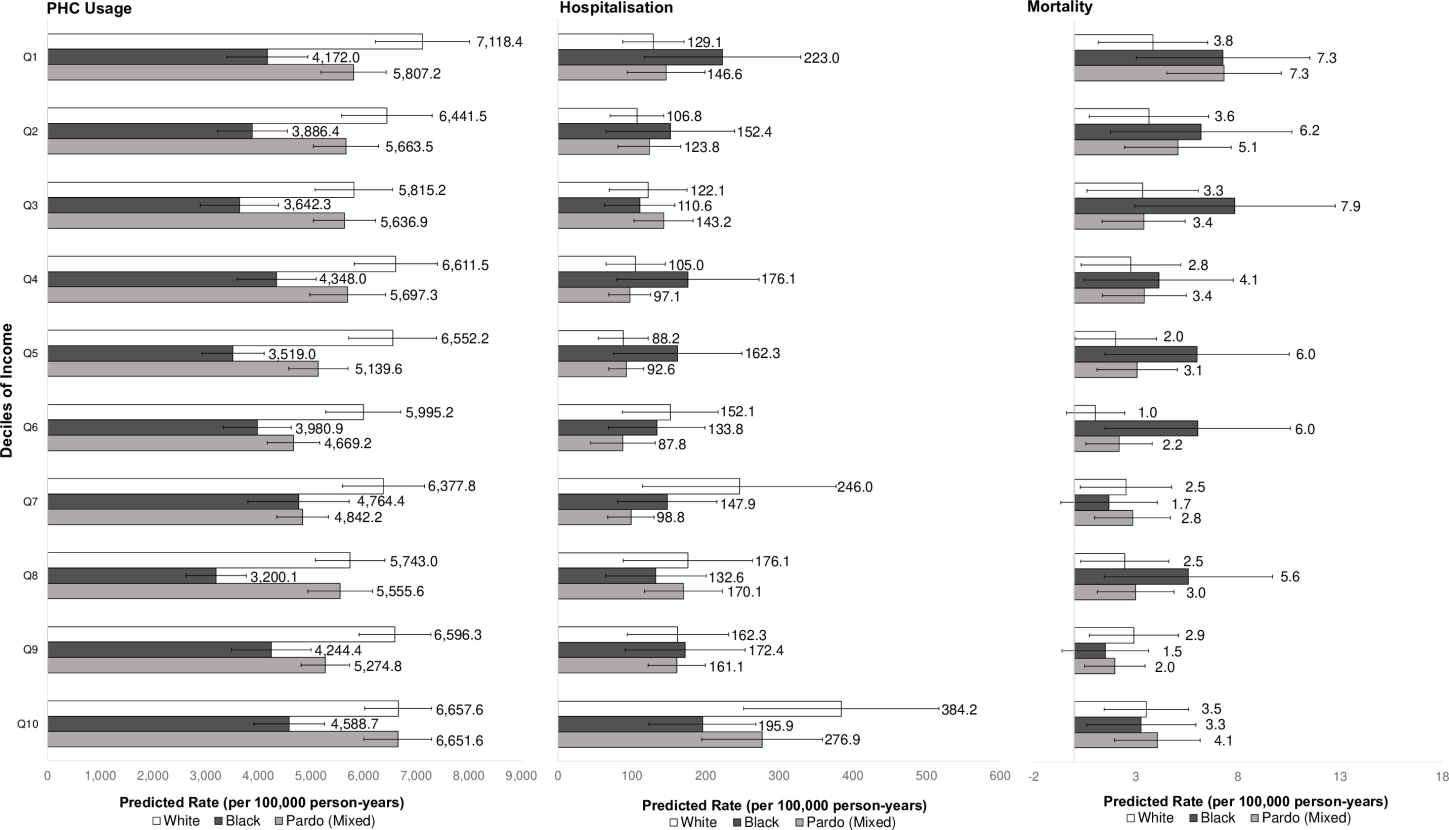
Predicted primary healthcare utilisation, hospitalisation, and mortality rates per 100,000 person-years based on adjusted Poisson regression models for interactions between race/ethnicity and deciles of income with 95% confidence intervals displayed.Obtained from separate fully adjusted Poisson regressions per outcome (PHC utilisation [registered users only], hospitalisation, and mortality); adjusted for sex, education level, age group, disability, unemployment, number of family members per bedroom, household flooring, household piped water access, formal employment in the family, Bolsa Família-receiving family, quintiles of household expenditure on medicines and food. Results for individuals of other racial/ethnic groups were either indeterminate or inconsistent, therefore not included.

Interactions between race/colour and income were statistically significant for all outcomes (figure 3; online supplemental material 7) showing black and pardo individuals across all income deciles (Q1–Q10) had lower predicted PHC usage compared with the poorest (Q1) white individuals. Black and pardo individuals had higher predicted rates of hospitalisation at lower incomes, whereas white individuals generally had higher predicted rates of hospitalisation in the upper-income deciles. Similar to education, the negative effects of low income and black or pardo race/colour compounded to increase the risk of death for mental disorders. Black, and to some extent pardo individuals had higher predicted mortality rates than white individuals—particularly at lower incomes (online supplemental material 8). Interactions between race/colour and sex were not statistically significant (online supplemental material 9).

10.1136/bmjgh-2023-013327.supp7 Supplementary data

10.1136/bmjgh-2023-013327.supp8 Supplementary data

10.1136/bmjgh-2023-013327.supp9 Supplementary data

## Discussion

This study assessed the racial/colour inequalities in PHC usage, hospitalisation and mortality for mental health disorders in a cohort of 1.2 million low-income individuals in Rio de Janeiro between 2010 and 2016. Racial/colour inequalities in mental health outcomes were generally large, with black and pardo individuals having the lowest healthcare usage rates and highest levels of mortality. These racial/colour inequalities persisted after adjusting for a wide range of socioeconomic factors. Males, black and pardo individuals, and those with lower income had the lowest PHC usage rates for mental health, whereas males, white individuals and those with higher income (of low-income individuals) had the highest hospitalisation rates. PHC usage was predominantly for anxiety and mood affective disorders, over two-thirds of hospitalisations were for psychotic syndromes, and nearly 90% of deaths were attributable to substance abuse/dependence disorders and suicide. Individuals of black race/colour, lower educational attainment and lower household income had the highest mortality rates from mental disorders. Black and pardo race/colour, and lower socioeconomic status (eg, lower education or low income) interacted to confer additional disadvantage in mental healthcare outcomes, particularly mortality.

The racial/colour inequalities in PHC usage identified in this study are highly concordant with international and Brazilian research demonstrating the lower healthcare usage for rates of Afro-descendants and darker-skinned individuals.^23–25 47–58^ Despite greater coverage of ESF for black and pardo Brazilians, barriers to accessing health services remain due to the uneven distribution of resources, limited health literacy and unconscious bias against non-white individuals in healthcare practice which leads to a lack of trust in healthcare professionals.^5 57^ This may explain the lower rates of PHC usage and hospitalisations observed in non-white individuals in this study. Evidence from health services in the USA, identified that white individuals were more likely than black individuals to get a psychotherapy appointment, and white males were twice as likely to get appointments compared with black males.^58^ A randomised cross-sectional study in the USA found that of patient callers offered primary care appointments, black and Hispanic callers were offered appointments further in the future despite identical insurance as their white counterparts.^59^ Similarly, the National Audit Office in England found that white patients found it easier to get an appointment within general practice compared with other ethnic groups across all health conditions.^60 61^ Other evidence suggests that structural racism can adversely affect mental health due to differential access to resources and services, and poor living conditions.^5 11 62–64^ Furthermore, experiences of discrimination can induce physiological and psychological reactions that can lead to adverse changes in mental health status.^62 63^ However, in this study, adjustment for PHC usage in hospitalisation and mortality outcomes did not change the estimates by racial/colour groups, suggesting that PHC usage was not a key determinant of racial/colour inequalities in mental health hospitalisations or mortality.

Limited Brazilian research exists regarding mental disorder hospitalisation and mortality by race/colour—one study in Salvador, Bahia found higher hospitalisation rates for black and pardo individuals compared with white, but there was a high level of missing data for race/colour.^32^ However, studies in high-income countries (HICs) document higher hospital admissions for mental disorders in non-white racial/ethnic groups.^65 66^ One potential explanation is that non-white individuals are more likely to end up in crisis care for poor mental health rather than accessing primary care, as reported in the United Kingdom and United States of America (USA).^65 67^ This is often due to reduced access to PHC, stigma, and distrust of health systems among non-white individuals compared with white individuals, leading to delayed treatment and therefore, increased risk of inpatient hospitalisation.^68^

In this study, black individuals were nearly 70% more likely to die from mental disorders than white individuals, a finding supported by a recent study in São Paulo where ‘unnatural’ causes of death, including suicide, were associated with non-white race/colour and substance-related disorders.^29^ This differs from HICs where White individuals with mental illnesses often have higher ‘unnatural’ mortality rates.^69 70^ The different findings between HICs and evidence from Brazil in relation to mental health hospitalisations and mortality may stem from multiple factors including persisting healthcare access issues beyond PHC, such as difficulties in receiving treatments and referrals to specialists, differences in quality of care, as well as substantially higher levels of deprivation and poverty for non-white individuals in Brazil.^56 71 72^

Socioeconomic status is highly interlinked with race and ethnicity and explains many, but not all of the inequalities across racial and ethnic groups. In many countries, darker-skinned individuals and ethnic minorities are more likely to be of lower socioeconomic status and exposed to chronic stressors and mental disorder risk factors than other racial/ethnic groups, such as financial insecurity, family instability and drug/alcohol consumption.^73–75^ However, this study found that racial/colour inequalities in mental health outcomes persisted after controlling for a wide range of socioeconomic variables, similar to other studies in both Brazil and HICs suggesting factors, other than socioeconomic status, explain these inequalities.^76–80^ Brazil has a long history of structural racism that reduces healthcare access for black, pardo and indigenous Brazilians.^5 11 64^ In the USA, internalised stigma and limited mental health literacy surrounding mental health is also linked to a lack of willingness to seek care for mental health and poor treatment adherence in racial/ethnic minorities.^71^ Differential treatment, either intentional or unintentional, by healthcare providers can reduce care quality for individuals of non-white race/ethnicity, leading to poorer mental health outcomes.^5 81^ US studies identify implicit bias in mental healthcare providers, with biased treatment recommendations, poorer communication and care quality.^72 82^ Further studies in the USA have shown that black and Latino patients have decreased confidence in mental health services and a lack of trust in healthcare professionals and treatment, contributing to lower service use.^83 84^ Aversion to mental health treatment has also been observed in Brazil, particularly for low-income individuals.^85^

This study identified that being of black or pardo race/colour interacted with measures of lower socioeconomic status (low income and education) to exacerbate poor PHC usage and mortality risks for mental health. These findings align with intersectionality theory and the multiple jeopardy hypothesis, whereby the negative effects of simultaneously belonging to multiple marginalised or disadvantaged groups is greater than the total negative effects of belonging to each social group.^86^ These predisposing socioeconomic factors form interlocking systems of oppression, particularly in Rio de Janeiro where income and gender inequality, violence and national issues associated with structural racism and limited employment and education opportunities are widespread.^5 11 87^ However, international studies, arguably in settings with lower racial/ethnic and social inequality, have shown contraindicating and inconsistent results regarding intersecting axes of race/colour inequalities in both a general health and mental health context.^88–93^ This warrants further research to strengthen the empirical evidence of intersectionality in healthcare and health outcomes.

There are key policy recommendations that stem from this work. There is a need to focus on the structural drivers of racial/ethnic inequalities in Rio de Janeiro, in Brazil, and internationally to identify factors amenable to intervention. One potential method to support black and pardo Brazilians is by driving Community Health Worker (CHW) engagement with mental healthcare in Brazil to improve trust in the health system and mental health services. CHWs play a crucial role in the Brazilian health system, particularly in lower-income communities, bridging health services and communities with consideration of socioeconomic, cultural, and racial and ethnic factors.^94 95^ Promoting access to mental health services across the city and country’s regions is also vital and identifying and addressing socioeconomic and cultural barriers to access. In Brazil, this means strengthening both healthcare and social assistance services as part of the *Rede de Atenção Psicossocial* (Psychosocial Care Network), which is a gateway for individuals with mental distress and disorders, particularly for those with substance abuse disorders.^96^ This underlies the importance of continued and equitable financial investment in mental healthcare, not only in Brazil, but globally. From the service perspective, there is a need to strengthen capacity of community-based mental healthcare to identify poor mental health, prioritising prevention and reducing costs to services, simultaneously tackling income inequalities in healthcare access.^97^ Overall, the findings indicate that policies must address mental disorder risk factors and the wider social determinants of mental health. This includes reinforcing protective mental health factors such as strengthening support networks and building social relationships, as well as improving access to education and employment to improve emotional and financial stability.^8 98^

A key strength of this study was the use of a dataset of 1.2 million individual individuals with linked health and healthcare data, and rich, detailed socioeconomic data. While linkage of datasets could have introduced bias, this was minimised by separately linking *Cadastro Único* to mortality datasets and primary care records, reducing interference. Further, linkage was determined as high quality with a 99% precision, as detailed elsewhere.^99^ This study used clinical diagnoses from electronic medical records (both ICD-10 and ICPC codes), However, under-reporting and under-diagnosis of mental disorders in LMICs is common,^1^ potentially underestimating the true mental disorder prevalence and as under-reporting is likely associated with race/ethnicity, inequalities may have been underestimated.^100^ Socioeconomic data was self-reported, potentially leading to misclassification^101^; however, this bias was unlikely to be sizeable enough or correlated with skin colour sufficiently to invalidate the findings.

Missing data for race/colour was reported as ‘other’, which could have affected the results, but this was less than 2.5% of individuals and also included individuals of *amarelo* (Asian) or Indigenous race/colour. Additionally, in recent years, there has been an increase in the number of Brazilians identifying as black/pardo and fewer identifying as white,^12 102^ which means that rates of PHC usage, hospitalisation and mortality could change which is important to acknowledge. The demographic profile of the cohort has implications for generalisability of the findings. The racial/colour distribution of the cohort is different to that of the general population of Rio de Janeiro, which may affect the generalisability of the results. In this study, white, black and pardo individuals represented 29.6%, 17.5% and 50.7% of the cohort, respectively and 29.3%, 17.6% and 51.1% of those ESF-registered. Yet in the city of Rio de Janeiro in 2010, white, black and pardo individuals represented 51.2%, 11.5% and 26.5% of the population, respectively.^13^ Furthermore, the cohort was composed of individuals claiming government welfare through the *Cadastro Único*. Welfare recipients often face significant socioeconomic disadvantage, barriers to healthcare access, financial instability and food insecurity which contributes to poorer mental health outcomes and can exacerbate mental disorder risk.^8^ However, low-income populations also face challenges in accessing high-quality healthcare and may not receive mental health diagnoses. The findings of this study should not be directly extrapolated to the general population without caution.

There are other limitations from a lack of data from other mental healthcare services in the city. Although this study addressed multiple mental disorders, PHC usage for mental health was predominantly for common mental disorders including depression and anxiety, whereas individuals with more severe mental disorders would be most likely to access the specialist *Centros de Atenção Psicossocial* (Psychosocial Care Centres; CAPS). Unfortunately, data from CAPS services was not available. Therefore, the hospitalisation and mortality estimates exclude conditions diagnosed/treated in certain specialist services, meaning the true burden is likely to be underestimated. Finally, this study did not adjust for prior hospitalisation and family history of mental disorders, or violence which may affect pathways to healthcare seeking or exacerbate mental disorder risk factors, and the results should be interpreted with appropriate caution.

While the specific historical and cultural factors driving racial/colour and ethnic inequalities in Brazil may differ from those in other countries, this study provides important insights that can be applied globally. Racial discrimination is not limited to Brazil; populations such as migrants, ethnic minorities and Indigenous Peoples face discrimination worldwide. Racial/ethnic disparities are widespread and unjust in many countries, with common underlying causes. While much of the evidence on inequalities comes from the USA and Europe, there is a pressing need for research from other parts of the world, particularly from LMICs and in marginalised areas like *favelas*. This study contributes to a better understanding of the root causes and magnitude of mental health inequalities and can serve as a platform for similar research in diverse settings, fostering a global dialogue on addressing these inequalities.

## Conclusion

Inequalities in mental healthcare usage and mental health outcomes were large and not fully explainable by socioeconomic factors in low-income individuals in Rio de Janeiro, Brazil. Black and pardo Brazilians had lower healthcare usage and black Brazilians had higher mortality rates for mental disorders. Overall, black and pardo Brazilians experienced worse mental health outcome when part of low-income and low-education groups.

10.1136/bmjgh-2023-013327.supp10 Supplementary data

## Data Availability

The datasets analysed in this study were generated via linkage of routine healthcare and administrative datasets. Publicly available datasets are available from Brazilian government websites: http://tabnet.datasus.gov.br/ and https://www.gov.br/pt-br/servicos/solicitar-cessao-de-dados-identificados-do-cadastro-unico. The specific datasets used in this analysis (individual-level records with names and tax numbers obtained following linkage) were obtained from the Secretariat for Health in Rio de Janeiro. These linked datasets, analysed in this study, are not publicly available due the confidentiality and sensitivity of the linked individual-level data. However, the corresponding author is available to assist other researchers requesting approval from Brazilian authorities and obtaining appropriate ethical approval for re-use of these datasets.
