# Supplementary material for: Racial inequalities in mental healthcare use and mortality: a cross-sectional analysis of 1.2 million low-income individuals in Rio de Janeiro, Brazil 2010–2016
Source: BMJ Glob Health. 2023 Dec 2;8(12):e013327. doi: 10.1136/bmjgh-2023-013327 (PMC10693873; doi:10.1136/bmjgh-2023-013327)
Supplement: Supplementary data [file bmjgh-2023-013327supp001.pdf]

Supplemental Material 1 | Overview of Data Sources & Variables.
Adapted from Hone et al. 2020 *Primary healthcare expansion and mortality in Brazil's urban poor: A cohort analysis of 1.2 million adults.*

Database	Overview	Source	Time Period	Key Variables
Cadastro Único	A national administrative database on all individuals who are claiming government welfare. Contains 1.2 million adults aged 15 years or older.	*Secretaria Municipal de Assistência Social* (Municipal Secretariat of Social Assistance; SMAS)	2015 extraction (all individuals registered before 1st January 2015)	***Individual-level Variables***<br>Sex (male, female); race/colour (black, white, pardo (mixed race), other); age at cohort entry in years (15-17, 18-19, 20-22, 23-24, 25-29, 30-34, 35-39, 40-44, 45-49, 50-59, 60-69, 70 or more); highest level of education (none/preschool/literacy class, elementary school, high school or higher education); disability (yes, no); unemployed at cohort entry (yes, no).<br><br>***Household-level Variables***<br>Household per capita income decile; number of family members per bedroom (two or fewer, more than 2, 3 or fewer, more than 3, 4 or fewer, more than 4); household flooring (cement, wood, ceramic, tiles, other); household piped water access (yes, no); formal employment in the family (yes, no); *Bolsa Família*-receiving family (yes, no); quintiles of household expenditure on medicines; quintiles of per capita household expenditure on food.
ESF Electronic health records (EHR)	A municipal dataset containing dates of individuals' registration with ESF clinics and the dates of their contacts with health professionals.	Secretaria Municipal de Saúde (Municipal Health Secretariat; SMS)	1st January 2010 to 31st December 2016	Total primary healthcare consultations (ICPC codes).
Sistema de Informações sobre Mortalidade (the Mortality Information System; SIM)	National universal dataset of all death certificates	SMS	1st January 2010 to 31st December 2016	Mental health-associated cause of death (ICD-10 codes).
Sistema de Informações Hospitalares (Hospitalisation Information System; SIH)	National dataset of all hospital records for the public health system.	SMS	1st January 2010 to 31st December 2016	Total mental health-associated hospitalisations (ICD-10 and ICPC codes).
