# Supplementary material for: Racial inequalities in mental healthcare use and mortality: a cross-sectional analysis of 1.2 million low-income individuals in Rio de Janeiro, Brazil 2010–2016
Source: BMJ Glob Health. 2023 Dec 2;8(12):e013327. doi: 10.1136/bmjgh-2023-013327 (PMC10693873; doi:10.1136/bmjgh-2023-013327)
Supplement: Supplementary data [file bmjgh-2023-013327supp002.pdf]

Supplemental Material 2 | Mental Disorder Disaggregation by International Classification of Disease (ICD-10) & International Classification of Primary Care (ICPC) Codes.

Mental Disorder Classification	ICD-10 Codes	ICPC Codes & Definitions
Substance Abuse/Dependence Disorders<br>Mental and behavioural disorders due to use of alcohol<br>Mental and behavioural disorders due to use of opioids<br>Mental and behavioural disorders due to use of cannabinoids<br>Mental and behavioural disorders due to use of sedatives or hypnotics<br>Mental and behavioural disorders due to use of cocaine<br>Mental and behavioural disorders due to use of other stimulants, including caffeine<br>Mental and behavioural disorders due to use of hallucinogens<br>Mental and behavioural disorders due to use of tobacco<br>Mental and behavioural disorders due to use of volatile solvents<br>Mental and behavioural disorders due to multiple drug use and use of other psychoactive substances	F10-F19	P15 Chronic alcohol abuse<br>P17 Tobacco abuse<br>P18 Medication abuse<br>P19 Drug abuse
Psychotic Syndromes<br>Schizophrenia<br>Schizotypal disorder<br>Persistent delusional disorders<br>Acute and transient psychotic disorders<br>Induced delusional disorder<br>Schizoaffective disorders<br>Other nonorganic psychotic disorders<br>Unspecified nonorganic psychosis	F20-F25, F28, F29	P29 Psychological symptom/complt other<br>P71 Organic psychosis disorder<br>P72 Schizophrenia<br>P73 Affective psychosis<br>P98 Psychosis NOS/other<br>P99 Psychological disorders, other
Mood Affective Disorders<br>Manic episode<br>Bipolar affective disorder<br>Depressive episode<br>Recurrent depressive disorder<br>Persistent mood affective disorder<br>Other mood affective disorders<br>Unspecified mood affective disorder	F30-F34, F38, F39	P03 Feeling depressed<br>P76 Depressive disorder

(Continued)

Supplemental Material 2 | *(Continued).*

Neurotic, Stress-related & Somatoform Disorders (Anxiety Disorders)<br>Phobic anxiety disorders<br>Other anxiety disorders<br>Obsessive-compulsive disorder<br>Reaction to severe stress, and adjustment disorders<br>Dissociative conversion disorders<br>Somatoform disorders<br>Other neurotic disorders	F40-F45, F48	P01 Feeling anxious/nervous/tense<br>P02 Acute stress reaction<br>P74 Anxiety disorder/anxiety state<br>P79 Phobia/compulsive disorder<br>P82 Post-traumatic stress disorder
Personality & Behaviour Disorders<br>Specific personality disorders<br>Mixed and other personality disorders<br>Enduring personality changes, not attributable to brain damage and disease<br>Habit and impulse disorders<br>Conduct disorders<br>Mixed disorders of conduct and emotions<br>Emotional disorders with onset specific to childhood<br>Disorders of social functioning with onset specific to childhood and adolescence<br>Eating disorders<br>Nonorganic sleep disorders	F50, F51, F60-F63, F91-F94	P80 Personality disorder<br>P86 Anorexia nervosa/bulimia
Suicide/Associated Outcomes<br>Intentional self-poisoning<br>Intentional self-harm<br>Suicidal ideation	X60-X69, X70-X84, R45.851	P77 Suicide/suicide attempt
