# Supplementary material for: Racial inequalities in mental healthcare use and mortality: a cross-sectional analysis of 1.2 million low-income individuals in Rio de Janeiro, Brazil 2010–2016
Source: BMJ Glob Health. 2023 Dec 2;8(12):e013327. doi: 10.1136/bmjgh-2023-013327 (PMC10693873; doi:10.1136/bmjgh-2023-013327)
Supplement: Supplementary data [file bmjgh-2023-013327supp003.pdf]

Desigualdades raciais no uso de saúde mental e mortalidade: uma análise transversal de 2010-2016 de 1,2 milhão de indivíduos de baixa renda no Rio de Janeiro, Brasil

RESUMO

Introdução

Desigualdades na saúde mental entre grupos raciais são enormes e injustos em diversos países, e ainda assim essas desigualdades são pouco investigadas, particularmente nos países de baixa e média renda como o Brasil.

Métodos

Realizou-se um estudo transversal de 1,2 milhões de adultos de baixa renda no Rio de Janeiro, Brasil, com dados socioeconômicos, saúde mental, e mortalidade. Modelos de regressão de Poisson foram usados para investigar as associações entre raça/cor e utilização da atenção primária à saúde (APS), hospitalização, e mortalidade decorrente de doença mental, levando em conta diversos fatores socioeconômicos. Interações entre raça/etnicidade e características socioeconômicas (sexo, nível de educação, renda) foram examinadas se pessoas pretas e pardas enfrentaram riscos agravados por resultados desfavoráveis à saúde mental.

Resultados

Houve 272.532 consultas na APS, 10.970 hospitalizações, e 259 mortes decorrentes de doenças mentais no período de 2010 a 2016. Após ajuste para fatores socioeconômicos, a menor taxa de utilização da APS foi observada em pessoas pretas (razão de taxas ajustadas [RTA]:0,64; IC95%:0,60–0,68; comparado com brancos) e pessoas pardas (RTA:0,87; IC95%:0,83–0,92;), e homens. Pessoas pretas eram mais suscetíveis a morrer de doenças mentais (RTA:1,68; IC95%:1,19–2,37; comparado com brancos), como aqueles com nível de educação mais baixo e baixa renda familiar. Em modelos de interação, ser preto ou pardo confere desvantagem adicional nos desfechos de saúde mental. Pessoas pretas (RTA:0,56; IC95%:0,47–0,66) e pardos (RTA:0,75; IC95%:0,66–0,87), com o mais alto nível de educação, tem a mais baixa taxa de utilização da APS para doenças mentais, quando comparados com pessoas brancas com o menor nível de educação. Pessoas pretas tinham uma probabilidade 3,7 vezes maior (RTA:3,67; IC95%:1,29–10,42) de morrer de doenças mentais, se comparados com pessoas brancas com o mesmo nível de educação.

Conclusão

Para indivíduos de baixa renda no Rio de Janeiro, desigualdades raciais dos desfechos de saúde mental eram enormes e não completamente justificadas pelas condições socioeconômicas. Brasileiros pretos e pardos eram consistentemente negativamente afetados, com baixa utilização da APS e piores desfechos na saúde mental.
