# Supplementary material for: Racial inequalities in mental healthcare use and mortality: a cross-sectional analysis of 1.2 million low-income individuals in Rio de Janeiro, Brazil 2010–2016
Source: BMJ Glob Health. 2023 Dec 2;8(12):e013327. doi: 10.1136/bmjgh-2023-013327 (PMC10693873; doi:10.1136/bmjgh-2023-013327)
Supplement: Supplementary data [file bmjgh-2023-013327supp004.pdf]

Supplemental Material 4 | Adjusted rates ratios from Poisson regression models of mental disorder associated hospitalisations and mortality by all sociodemographic variables and PHC use for mental disorders.

	Hospitalisation		Hospitalisation+		Mortality		Mortality+	
**Characteristics**	**ARR**	**ARR**	**ARR**	**95% CI**	**ARR**	**95% CI**	**ARR**	**95% CI**
**Individual**								
**Sex**								
Male	1 (ref)	–	1 (ref)	–	1 (ref)	–	1 (ref)	–
Female	0.57***	(0.49–0.67)	0.54***	(0.46–0.64)	0.32***	(0.25–0.42)	0.32***	(0.25–0.42)
**Race/Colour**								
White	1 (ref)	–	1 (ref)	–	1 (ref)	–	1 (ref)	–
Black	0.93	(0.75–1.15)	0.99	(0.80–1.22)	1.68**	(1.19–2.37)	1.68**	(1.19–2.38)
Pardo (Mixed)	0.83*	(0.70–0.99)	0.85	(0.71–1.01)	1.27	(0.94–1.73)	1.28	(0.94–1.73)
Other	0.55**	(0.37–0.83)	0.58**	(0.38–0.87)	0.78	(0.29–2.19)	0.79	(0.28–2.20)
**Education Level**								
None/Preschool/Literacy Class	1 (ref)	–	1 (ref)	–	1 (ref)	–	1 (ref)	–
Elementary	0.92	(0.70–1.22)	0.93*	(0.70–1.23)	0.86	(0.60–1.24)	0.87	(0.60–1.25)
High School or Higher Education	0.78	(0.57–1.07)	0.80***	(0.58–1.10)	0.53**	(0.34–0.85)	0.55	(0.34–0.86)
**Age Group (years)**								
15-19	1 (ref)	–	1 (ref)	–	1 (ref)	–	1 (ref)	–
20-22	3.09***	(2.14–4.46)	3.07***	(2.13–4.43)	3.63**	(1.52–8.66)	3.62**	(1.52–8.66)
23-24	4.71***	(3.18–6.97)	4.65***	(3.14–6.89)	6.46***	(2.71–15.43)	6.46***	(2.71–15.43)
25-29	6.19***	(4.29–8.93)	6.01***	(4.17–8.66)	4.97***	(2.14–11.52)	4.96***	(2.14–11.51)
30-34	8.94***	(6.02–13.29)	8.29***	(5.59–12.30)	5.69***	(2.36–13.67)	5.67***	(2.36–13.63)
35-39	8.98***	(6.21–13.00)	8.21***	(5.66–11.91)	5.48***	(2.27–13.20)	5.46***	(2.27–13.13)
40-44	9.28***	(6.16–13.97)	8.32***	(5.51–12.57)	11.03***	(4.96–24.54)	10.97***	(4.93–24.42)
45-49	6.34***	(4.32–9.29)	5.72***	(3.90–8.38)	9.07***	(3.98–20.68)	9.02***	(3.96–20.54)
50-59	5.83***	(3.95–8.59)	5.21***	(3.53–7.68)	13.32***	(6.15–28.85)	13.22***	(6.10–28.66)
60-69	2.27***	(1.43–3.58)	2.18**	(1.38–3.45)	17.96***	(8.06–40.06)	17.86**	(8.01–39.83)
70+	1.42	(0.70–2.89)	1.57	(0.77–3.18)	19.56***	(7.96–48.06)	19.57**	(7.97–48.05)
**Disability**								
No	1 (ref)	–	1 (ref)	–	1 (ref)	–	1 (ref)	–
Yes	13.71***	(11.42–16.46)	10.90***	(8.98–13.23)	1.73*	(1.05–2.86)	1.71*	(1.04–2.81)
**Unemployed**								
No	1 (ref)	–	1 (ref)	–	1 (ref)	–	1 (ref)	–
Yes	2.11***	(1.78–2.49)	1.95***	(1.65–2.32)	1.19	(0.86–1.66)	1.19	(0.85–1.66)

Continued

Supplemental Material 4 | (*Continued*).

	Hospitalisation		Hospitalisation+		Mortality		Mortality+	
**Characteristics**	**ARR**	**ARR**	**ARR**	**95% CI**	**ARR**	**95% CI**	**ARR**	**95% CI**
**Household**								
**Deciles of Income**								
Q1 (Poorest)	1 (ref)	–	1 (ref)	–	1 (ref)	–	1 (ref)	–
Q2	0.79	(0.57–1.08)	0.75	(0.55–1.03)	0.77	(0.49–1.22)	0.77	(0.48–1.22)
Q3	0.82	(0.60–1.11)	0.78	(0.57–1.05)	0.67	(0.42–1.08)	0.67	(0.42–1.07)
Q4	0.72*	(0.53–0.99)	0.71*	(0.52–0.97)	0.56*	(0.34–0.92)	0.56*	(0.34–0.92)
Q5	0.65**	(0.48–0.89)	0.64**	(0.47–0.87)	0.52*	(0.31–0.87)	0.52*	(0.31–0.87)
Q6	0.72	(0.50–1.04)	0.71	(0.49–1.01)	0.40**	(0.22–0.72)	0.40**	(0.22–0.72)
Q7	0.96	(0.65–1.41)	0.94	(0.64–1.38)	0.40**	(0.22–0.71)	0.40**	(0.22–0.71)
Q8	1.02	(0.71–1.46)	0.99	(0.69–1.41)	0.55*	(0.33–0.92)	0.55*	(0.33–0.91)
Q9	1.03	(0.73–1.45)	0.98	(0.69–1.38)	0.35**	(0.19–0.64)	0.35**	(0.19–0.64)
Q10 (Richest)	1.84**	(1.25–2.70)	1.75**	(1.19–2.56)	0.60	(0.35–1.01)	0.60	(0.35–1.01)
**Bolsa Família-Claiming Family**								
No	1 (ref)	–	1 (ref)	–	1 (ref)	–	1 (ref)	–
Yes	1.22*	(1.00–1.49)	1.18	(0.97–1.43)	1.18	(0.87–1.60)	1.18	(0.87–1.60)
**Family Members per Bedroom**								
2 or fewer	1 (ref)	–	1 (ref)	–	1 (ref)	–	1 (ref)	–
more than 2, 3 or fewer	0.68***	(0.55–0.83)	0.68***	(0.55–0.84)	1.16	(0.84–1.61)	1.16	(0.84–0.00)
more than 3, 4 or fewer	0.61***	(0.48–0.79)	0.62***	(0.48–0.81)	0.94	(0.64–1.39)	0.94	(0.64–0.00)
more than 4	0.67*	(0.49–0.93)	0.67*	(0.49–0.93)	1.00	(0.68–1.48)	1.00	(0.68–0.00)
**Household Flooring Material**								
Soil	1 (ref)	–	1 (ref)	–	1 (ref)	–	1 (ref)	–
Cement	0.51***	(0.40–0.65)	0.51***	(0.40–0.65)	1.00	(0.66–1.52)	1.00	(0.66–1.51)
Repurposed Wood	0.76	(0.49–1.17)	0.76	(0.50–1.17)	1.11	(0.43–2.85)	1.10	(0.43–2.84)
Ceramics/Tiles	0.49***	(0.39–0.62)	0.49***	(0.39–0.62)	0.96	(0.65–1.41)	0.95	(0.65–1.40)
Other	1.22	(0.79–1.86)	1.25	(0.81–1.92)	2.05*	(1.03–4.05)	2.04*	(1.03–4.03)
**Piped Water Access**								
No	1 (ref)	–	1 (ref)	–	1 (ref)	–	1 (ref)	–
Yes	0.73	(0.48–1.12)	0.75	(0.49–1.15)	0.63	(0.37–1.09)	0.63	(0.37–1.09)
**Formal Employment in the Family**								
No	1 (ref)	–	1 (ref)	–	1 (ref)	–	1 (ref)	–
Yes	0.76*	(0.59–0.96)	0.78*	(0.61–0.99)	0.98	(0.65–1.46)	0.98	(0.65–1.46)

Continued

Supplemental Material 4 | (*Continued*).

	Hospitalisation		Hospitalisation+		Mortality		Mortality+	
**Characteristics**	**ARR**	**ARR**	**ARR**	**95% CI**	**ARR**	**95% CI**	**ARR**	**95% CI**
**Household**								
**Quintiles of per capita Expenditure on Medicines**								
Q1 (Least)	1 (ref)	–	1 (ref)	–	1 (ref)	–	1 (ref)	–
Q2	1.07	(0.77–1.48)	1.05	(0.76–1.45)	1.27	(0.81–2.00)	1.27	(0.80–2.00)
Q3	0.88	(0.66–1.18)	0.88	(0.65–1.17)	0.88	(0.48–1.63)	0.88	(0.48–1.63)
Q4	0.76	(0.54–1.07)	0.74	(0.52–1.04)	1.40	(0.77–2.54)	1.40	(0.77–2.53)
Q5 (Most)	0.71	(0.49–1.04)	0.70	(0.48–1.01)	1.34	(0.70–2.57)	1.34	(0.70–2.56)
**Quintiles of per capita Expenditure on Food**								
Q1 (Least)	1 (ref)	–	1 (ref)	–	1 (ref)	–	1 (ref)	–
Q2	0.57***	(0.46–0.71)	0.59***	(0.47–0.72)	0.66*	(0.46–0.95)	0.66*	(0.46–0.95)
Q3	0.62***	(0.50–0.78)	0.64***	(0.51–0.81)	0.62*	(0.42–0.92)	0.62*	(0.42–0.92)
Q4	0.58***	(0.45–0.75)	0.60***	(0.46–0.77)	0.58*	(0.38–0.88)	0.58*	(0.38–0.88)
Q5 (Most)	0.53***	(0.40–0.70)	0.55***	(0.42–0.73)	0.56*	(0.36–0.88)	0.56*	(0.36–0.88)
**PHC Use for Mental Disorders**								
No	–	–	1 (ref)	–	–	–	1 (ref)	–
Yes	–	–	5.86***	(4.82–7.11)	–	–	1.29	(0.68–2.45)
Total Observations (N)	*1,243,932*		*1,243,932*		*1,243,932*		*1,243,932*	

PHC – Primary Healthcare; ARR – Adjusted Rate Ratios; 95% CI – 95% Confidence Intervals.
Separate fully adjusted Poisson regressions per outcome (PHC usage, hospitalisation, and mortality); adjusted for sex, race/colour, education level, age group, disability, unemployment, household per capita income decile, number of family members per bedroom, household flooring, household piped water access, formal employment in the family, Bolsa Família-receiving family, quintiles of household expenditure on medicines and food.
+Adjusted for PHC use for mental disorders.
*Robust standard errors. *p<0.05; **p<0.01; *** p<0.001.*
