# Supplementary material for: Racial inequalities in mental healthcare use and mortality: a cross-sectional analysis of 1.2 million low-income individuals in Rio de Janeiro, Brazil 2010–2016
Source: BMJ Glob Health. 2023 Dec 2;8(12):e013327. doi: 10.1136/bmjgh-2023-013327 (PMC10693873; doi:10.1136/bmjgh-2023-013327)
Supplement: Supplementary data [file bmjgh-2023-013327supp005.pdf]

Supplemental Material 5 | Complete Poisson regression results from interactions between race/colour and education level.

	PHC usage		Hospitalisation		Mortality	
**Characteristics**	**ARR**	**95% CI**	**ARR**	**95% CI**	**ARR**	**95% CI**
**Individual**						
**Sex**						
Male	1 (ref)	–	1 (ref)	–	1 (ref)	–
Female	1.80***	(1.70–1.90)	0.58***	(0.49–0.67)	0.32***	(0.25–0.42)
**Race/Colour**						
White	1 (ref)	–	1 (ref)	–	1 (ref)	–
Black	0.76**	(0.62–0.93)	0.78	(0.39–1.56)	3.67*	(1.29–10.42)
Pardo (Mixed)	0.97	(0.81–1.16)	0.71	(0.38–1.35)	1.87	(0.69–5.09)
Other	0.56***	(0.43–0.73)	0.31**	(0.13–0.74)	1.17	(0.22–6.09)
**Education Level**						
None/Preschool/Literacy Class	1 (ref)	–	1 (ref)	–	1 (ref)	–
Elementary	0.94	(0.82–1.08)	0.76	(0.43–1.33)	1.35	(0.53–3.41)
High School or Higher Education	0.88	(0.76–1.01)	0.76	(0.43–1.36)	0.99	(0.35–2.79)
**Age Group (years)**						
15-19	1 (ref)	–	1 (ref)	–	1 (ref)	–
20-22	1.54***	(1.33–1.78)	3.09***	(2.14–4.45)	3.65**	(1.53–8.72)
23-24	2.11***	(1.76–2.52)	4.71***	(3.18–6.97)	6.50***	(2.72–15.53)
25-29	2.99***	(2.64–3.38)	6.21***	(4.30–8.96)	5.00***	(2.15–11.60)
30-34	5.04***	(4.48–5.66)	8.95***	(6.02–13.30)	5.72***	(2.38–13.76)
35-39	6.87***	(6.16–7.66)	8.99***	(6.21–13.02)	5.50***	(2.28–13.25)
40-44	8.43***	(7.57–9.39)	9.29***	(6.17–14.00)	11.07***	(4.97–24.64)
45-49	9.98***	(8.95–11.14)	6.36***	(4.33–9.32)	9.10***	(3.99–20.76)
50-59	11.12***	(10.04–12.31)	5.84***	(3.96–8.61)	13.36***	(6.16–28.96)
60-69	10.03***	(8.92–11.28)	2.27***	(1.44–3.60)	18.01***	(8.07–40.18)
70+	5.70***	(4.92–6.61)	1.43	(0.71–2.92)	19.54***	(7.95–48.04)
**Disability**						
No	1 (ref)	–	1 (ref)	–	1 (ref)	–
Yes	2.96***	(2.75–3.17)	13.66***	(11.37–16.40)	1.73*	(1.05–2.86)
**Unemployed**						
No	1 (ref)	–	1 (ref)	–	1 (ref)	–
Yes	1.61***	(1.52–1.69)	2.10***	(1.77–2.49)	1.19	(0.86–1.67)
**Household**						
**Deciles of Income**						
Q1 (Poorest)	1 (ref)	–	1 (ref)	–	1 (ref)	–
Q2	0.95	(0.86–1.06)	0.79	(0.57–1.08)	0.77	(0.49–1.22)
Q3	0.91	(0.82–1.01)	0.82	(0.60–1.10)	0.67	(0.42–1.08)
Q4	0.98	(0.88–1.09)	0.72*	(0.53–0.99)	0.56*	(0.34–0.92)
Q5	0.90*	(0.81–1.00)	0.65**	(0.48–0.89)	0.52*	(0.31–0.87)
Q6	0.85**	(0.76–0.94)	0.72	(0.50–1.04)	0.40**	(0.22–0.72)
Q7	0.91	(0.82–1.01)	0.96	(0.66–1.41)	0.40**	(0.22–0.72)
Q8	0.88*	(0.79–0.98)	1.02	(0.71–1.46)	0.55*	(0.33–0.91)
Q9	0.94	(0.85–1.04)	1.03	(0.72–1.45)	0.35**	(0.19–0.64)
Q10 (Richest)	1.07	(0.96–1.20)	1.83**	(1.25–2.69)	0.60	(0.35–1.01)

(Continued)

Supplementary Material 5 | (*Continued*).

Characteristics	PHC usage		Hospitalisation		Mortality	
	ARR	95% CI	ARR	95% CI	ARR	95% CI
**Household**						
**Bolsa Família-Claiming Family**						
No	1 (ref)	–	1 (ref)	–	1 (ref)	–
Yes	1.01	(0.95–1.07)	1.22*	(1.00–1.49)	1.18	(0.87–1.60)
**Family Members per Bedroom**						
2 or fewer	1 (ref)	–	1 (ref)	–	1 (ref)	–
more than 2, 3 or fewer	0.85***	(0.80–0.90)	0.68***	(0.55–0.00)	1.16	(0.84–1.61)
more than 3, 4 or fewer	0.76***	(0.71–0.81)	0.61***	(0.48–0.00)	0.94	(0.64–1.39)
more than 4						
**Household Flooring Material**						
Soil	1 (ref)	–	1 (ref)	–	1 (ref)	–
Cement	1.17***	(1.08–1.27)	0.51***	(0.40–0.65)	1.00	(0.66–1.52)
Repurposed Wood	1.09	(0.94–1.26)	0.75	(0.49–1.15)	1.10	(0.43–2.82)
Ceramics/Tiles	1.12**	(1.04–1.21)	0.49***	(0.39–0.62)	0.95	(0.65–1.40)
Other	1.05	(0.89–1.24)	1.21	(0.79–1.85)	2.03*	(1.02–4.03)
**Piped Water Access**						
No	1 (ref)	–	1 (ref)	–	1 (ref)	–
Yes	0.72***	(0.62–0.84)	0.74	(0.48–1.12)	0.63	(0.37–1.09)
**Formal Employment in the Family**						
No	1 (ref)	–	1 (ref)	–	1 (ref)	–
Yes	0.91**	(0.86–0.98)	0.76*	(0.59–0.96)	0.98	(0.65–1.46)
**Quintiles of per capita Expenditure on Medicines**						
Q1 (Least)	1 (ref)	–	1 (ref)	–	1 (ref)	–
Q2	1.12**	(1.04–1.21)	1.07	(0.77–1.48)	1.27	(0.80–2.00)
Q3	1.20***	(1.11–1.31)	0.89	(0.66–1.19)	0.89	(0.48–1.63)
Q4	1.31***	(1.18–1.44)	0.76	(0.54–1.07)	1.40	(0.77–2.54)
Q5 (Most)	1.43***	(1.28–1.59)	0.71	(0.49–1.03)	1.34	(0.70–2.57)
**Quintiles of per capita Expenditure on Food**						
Q1 (Least)	1 (ref)	–	1 (ref)	–	1 (ref)	–
Q2	0.80***	(0.75–0.86)	0.57***	(0.46–0.71)	0.66*	(0.46–0.95)
Q3	0.80***	(0.74–0.85)	0.62***	(0.50–0.78)	0.62*	(0.42–0.92)
Q4	0.80***	(0.74–0.86)	0.58***	(0.45–0.75)	0.58*	(0.38–0.88)
Q5 (Most)						
Total Observations (N)	*743,746*		*1,243,932*		*1,243,932*	
INTERACTIONS						
**Race/Colour × Education Level**						
Black × Elementary	0.82	(0.65–1.02)	1.41	(0.68–2.92)	0.40	(0.13–1.24)
Black × High School or Higher	0.84	(0.66–1.08)	0.83	(0.38–1.80)	0.46	(0.12–1.67)
Pardo (Mixed) × Elementary	0.89	(0.73–1.08)	1.26	(0.65–2.46)	0.71	(0.24–2.06)
Pardo (Mixed) × High School or Higher Education	0.89	(0.73–1.09)	1.09	(0.54–2.20)	0.48	(0.14–1.62)
Other × Elementary	1.84***	(1.31–2.58)	2.27	(0.82–6.28)	0.98	(0.11–8.61)
Other × High School or Higher Education	1.80**	(1.20–2.70)	1.80	(0.56–5.84)	0.00***	(0.00–0.00)
Total Observations (N)	*743,746*		*1,243,932*		*1,243,932*	
	**PHC usage⁺**		**Hospitalisation**		**Mortality**	
Overall Interaction Significance	***		–		***	

(Continued)

Supplementary Material 5 | (*Continued*).

	PHC usage		Hospitalisation		Mortality	
**Characteristics**	**ARR**	**95% CI**	**ARR**	**95% CI**	**ARR**	**95% CI**
**Race/Colour × Education Level** *Fully Expanded*						
White × None/Preschool/Literacy Class	1 (ref)	–	–	–	1 (ref)	–
White × Elementary	0.94	(0.82–1.08)	–	–	1.35	(0.53–3.41)
White × High School or higher Education	0.88	(0.76–1.01)	–	–	0.99	(0.35–2.79)
Black × None/Preschool/Literacy Class	0.76**	(0.62–0.93)	–	–	3.67*	(1.29–10.42)
Black × Elementary	0.58***	(0.50–0.68)	–	–	1.98	(0.78–4.99)
Black × High School or Higher Education	0.56***	(0.47–0.66)	–	–	1.67	(0.58–4.76)
Pardo (Mixed) × None/Preschool/Literacy Class	0.97	(0.81–1.16)	–	–	1.87	(0.69–5.09)
Pardo (Mixed) × Elementary	0.81**	(0.71–0.93)	–	–	1.78	(0.73–4.37)
Pardo (Mixed) × High School or Higher Education	0.75***	(0.66–0.87)	–	–	0.88	(0.32–2.40)
Other × None/Preschool/Literacy Class	0.56***	(0.43–0.73)	–	–	1.17	(0.22–6.09)
Other × Elementary	0.97	(0.76–1.23)	–	–	1.55	(0.30–7.99)
Other × High School or Higher Education	0.88	(0.64–1.22)	–	–	0.00***	(0.00–0.00)
Total Observations (N)	*743,746*		–		*1,243,932*	

PHC – Primary Healthcare; ARR – Adjusted Rate Ratios; 95% CI – 95% Confidence Intervals.
*Separate fully adjusted Poisson regressions per outcome (PHC usage [ESF registered users only], hospitalisation, and mortality); adjusted for sex, age group, disability, unemployment, household per capita income decile, number of family members per bedroom, household flooring, household piped water access, formal employment in the family, Bolsa Família-receiving family, quintiles of household expenditure on medicines and food. Robust standard errors. *p<0.05; **p<0.01; *** p<0.001.*
