# Supplementary material for: Racial inequalities in mental healthcare use and mortality: a cross-sectional analysis of 1.2 million low-income individuals in Rio de Janeiro, Brazil 2010–2016
Source: BMJ Glob Health. 2023 Dec 2;8(12):e013327. doi: 10.1136/bmjgh-2023-013327 (PMC10693873; doi:10.1136/bmjgh-2023-013327)
Supplement: Supplementary data [file bmjgh-2023-013327supp006.pdf]

Supplemental Material 6 | Predicted rates per 100,000 person-years based on adjusted Poisson regression models for interactions between race/colour and education level.

Characteristics	PHC usage		Hospitalisation		Mortality	
	Predicted Rate (per 100,000 person-years)	95% CI	Predicted Rate (per 100,000 person-years)	95% CI	Predicted Rate (per 100,000 person-years)	95% CI
**Race/Colour × Education Level**						
White × None/Preschool/Literacy Class	6,936.6***	(6,053.90–7,819.28)	–	–	2.4*	(0.29–4.45)
White × Elementary	6,528.2***	(6,220.62–6,835.79)	–	–	3.2***	(2.23–4.17)
White × High School or higher Education	6,073.7***	(5,697.13–6,450.34)	–	–	2.4***	(1.10–3.61)
Black × None/Preschool/Literacy Class	5,257.7***	(4,375.66–6,139.65)	–	–	8.7**	(3.70–13.71)
Black × Elementary	4,050.1***	(3,768.02–4,332.25)	–	–	4.7***	(3.27–6.10)
Black × High School or Higher Education	3,880.8***	(3,437.33–4,324.20)	–	–	4.0**	(1.68–6.23)
Pardo (Mixed) × None/Preschool/Literacy Class	6,719.6***	(5,823.92–7,615.27)	–	–	4.4***	(2.29–6.57)
Pardo (Mixed) × Elementary	5,627.5***	(5,390.15–5,864.88)	–	–	4.2***	(3.36–5.08)
Pardo (Mixed) × High School or Higher Education	5,236.1***	(4,932.23–5,540.04)	–	–	2.1***	(1.09–3.09)
Other × None/Preschool/Literacy Class	3,878.5***	(2,953.13–4,803.82)	–	–	2.8	(-1.13–6.67)
Other × Elementary	6,713.7***	(5,350.41–8,076.94)	–	–	3.7	(-1.42–8.77)
Other × High School or Higher Education	6,121.9***	(4,305.94–7,937.95)	–	–	0.0***	(0.00–0.00)

PHC – Primary Healthcare; 95% CI – 95% Confidence Intervals.
Obtained from separate fully adjusted Poisson regressions per outcome (PHC usage [ESF registered users only], hospitalisation, and mortality); adjusted for sex, age group, disability, unemployment, household per capita income decile, number of family members per bedroom, household flooring, household piped water access, formal employment in the family, Bolsa Família-receiving family, quintiles of household expenditure on medicines and food.
$^{*}p<0.05$; $^{**}p<0.01$; $^{***}p<0.001$.
