# Supplementary material for: Racial inequalities in mental healthcare use and mortality: a cross-sectional analysis of 1.2 million low-income individuals in Rio de Janeiro, Brazil 2010–2016
Source: BMJ Glob Health. 2023 Dec 2;8(12):e013327. doi: 10.1136/bmjgh-2023-013327 (PMC10693873; doi:10.1136/bmjgh-2023-013327)
Supplement: Supplementary data [file bmjgh-2023-013327supp007.pdf]

Supplemental Material 7 | Complete Poisson regression results from interactions between race/colour and deciles of income.

	PHC usage		Hospitalisation		Mortality	
**Characteristics**	**ARR**	**95% CI**	**ARR**	**95% CI**	**ARR**	**95% CI**
**Individual**						
**Sex**						
Male	1 (ref)	–	1 (ref)	–	1 (ref)	–
Female	1.79***	(1.70–1.90)	0.57***	(0.49–0.67)	0.32***	(0.25–0.42)
**Race/Colour**						
White	1 (ref)	–	1 (ref)	–	1 (ref)	–
Black	0.59***	(0.47–0.73)	1.73	(0.99–3.03)	1.90	(0.77–4.64)
Pardo (Mixed)	0.82*	(0.70–0.96)	1.14	(0.72–1.80)	1.91	(0.88–4.17)
Other	0.49**	(0.31–0.79)	0.82	(0.30–2.23)	0.87	(0.10–7.30)
**Education Level**						
None/Preschool/Literacy Class	1 (ref)	–	1 (ref)	–	1 (ref)	–
Elementary	0.88**	(0.80–0.96)	0.93	(0.70–1.24)	0.86	(0.60–1.24)
High School or Higher Education	0.82***	(0.75–0.91)	0.79	(0.58–1.09)	0.54**	(0.34–0.86)
**Age Group (years)**						
15-19	1 (ref)	–	1 (ref)	–	1 (ref)	–
20-22	1.53***	(1.33–1.77)	3.09***	(2.14–4.45)	3.63**	(1.52–8.66)
23-24	2.11***	(1.76–2.52)	4.70***	(3.18–6.96)	6.47***	(2.71–15.46)
25-29	2.98***	(2.64–3.38)	6.17***	(4.27–8.90)	4.97***	(2.14–11.53)
30-34	5.03***	(4.48–5.66)	8.88***	(5.97–13.19)	5.68***	(2.36–13.67)
35-39	6.86***	(6.15–7.65)	8.93***	(6.17–12.93)	5.46***	(2.26–13.16)
40-44	8.42***	(7.56–9.37)	9.24***	(6.14–13.93)	11.00***	(4.94–24.49)
45-49	9.96***	(8.92–11.12)	6.30***	(4.30–9.24)	9.07***	(3.98–20.69)
50-59	11.09***	(10.01–12.28)	5.82***	(3.95–8.58)	13.29***	(6.14–28.81)
60-69	10.02***	(8.91–11.27)	2.27**	(1.43–3.58)	17.92***	(8.03–40.00)
70+	5.74***	(4.95–6.66)	1.41	(0.69–2.86)	19.29***	(7.83–47.53)
**Disability**						
No	1 (ref)	–	1 (ref)	–	1 (ref)	–
Yes	2.96***	(2.76–3.18)	13.76***	(11.46–16.52)	1.74*	(1.06–2.86)
**Unemployed**						
No	1 (ref)	–	1 (ref)	–	1 (ref)	–
Yes	1.61***	(1.53–1.69)	2.11***	(1.78–2.50)	1.19	(0.86–1.66)
**Household**						
**Deciles of Income**						
Q1 (Poorest)	1 (ref)	–	1 (ref)	–	1 (ref)	–
Q2	0.90	(0.76–1.08)	0.83	(0.53–1.30)	0.95	(0.33–2.73)
Q3	0.82*	(0.69–0.97)	0.95	(0.55–1.61)	0.87	(0.30–2.50)
Q4	0.93	(0.78–1.10)	0.81	(0.50–1.33)	0.72	(0.24–2.20)
Q5	0.92	(0.77–1.10)	0.68	(0.42–1.12)	0.53	(0.16–1.76)
Q6	0.84*	(0.71–1.00)	1.18	(0.69–2.01)	0.27	(0.06–1.28)
Q7	0.90	(0.75–1.07)	1.91*	(1.02–3.56)	0.66	(0.21–2.02)
Q8	0.81*	(0.68–0.96)	1.36	(0.74–2.50)	0.64	(0.21–1.97)
Q9	0.93	(0.79–1.09)	1.26	(0.73–2.18)	0.76	(0.27–2.13)
Q10 (Richest)	0.94	(0.79–1.10)	2.98***	(1.79–4.95)	0.91	(0.36–2.33)

(Continued)

Supplemental Material 7 | (*Continued).*

	PHC usage		Hospitalisation		Mortality	
**Characteristics**	**ARR**	**95% CI**	**ARR**	**95% CI**	**ARR**	**95% CI**
**Household**						
**Bolsa Família-Claiming Family**						
No	1 (ref)	–	1 (ref)	–	1 (ref)	–
Yes	1.01	(0.95–1.07)	1.23*	(1.01–1.50)	1.18	(0.87–1.61)
**Family Members per Bedroom**						
2 or fewer	1 (ref)	–	1 (ref)	–	1 (ref)	–
more than 2, 3 or fewer	0.85***	(0.80–0.90)	0.68***	(0.55–0.00)	1.16	(0.84–1.61)
more than 3, 4 or fewer	0.76***	(0.70–0.81)	0.61***	(0.48–0.00)	0.94	(0.64–1.39)
more than 4	0.83***	(0.76–0.90)	0.67*	(0.48–0.00)	1.00	(0.67–1.47)
**Household Flooring Material**						
Soil	1 (ref)	–	1 (ref)	–	1 (ref)	–
Cement	1.18***	(1.09–1.28)	0.51***	(0.40–0.65)	0.99	(0.65–1.51)
Repurposed Wood	1.10	(0.95–1.27)	0.75	(0.48–1.15)	1.09	(0.42–2.81)
Ceramics/Tiles	1.13**	(1.05–1.21)	0.49***	(0.39–0.62)	0.95	(0.65–1.39)
Other	1.06	(0.90–1.25)	1.21	(0.79–1.86)	2.03*	(1.02–4.01)
**Piped Water Access**						
No	1 (ref)	–	1 (ref)	–	1 (ref)	–
Yes	0.72***	(0.62–0.84)	0.74	(0.48–1.12)	0.63	(0.37–1.09)
**Formal Employment in the Family**						
No	1 (ref)	–	1 (ref)	–	1 (ref)	–
Yes	0.91**	(0.86–0.97)	0.76*	(0.59–0.97)	0.98	(0.66–1.47)
**Quintiles of per capita Expenditure on Medicines**						
Q1 (Least)	1 (ref)	–	1 (ref)	–	1 (ref)	–
Q2	1.12**	(1.04–1.20)	1.07	(0.77–1.48)	1.27	(0.80–2.01)
Q3	1.20***	(1.11–1.31)	0.88	(0.66–1.18)	0.89	(0.48–1.63)
Q4	1.30***	(1.18–1.44)	0.76	(0.54–1.07)	1.40	(0.77–2.53)
Q5 (Most)	1.43***	(1.28–1.60)	0.70	(0.48–1.02)	1.31	(0.69–2.51)
**Quintiles of per capita Expenditure on Food**						
Q1 (Least)	1 (ref)	–	1 (ref)	–	1 (ref)	–
Q2	0.80***	(0.75–0.86)	1.07	(0.46–0.70)	0.66*	(0.46–0.95)
Q3	0.79***	(0.74–0.85)	0.88	(0.49–0.78)	0.62*	(0.42–0.92)
Q4	0.80***	(0.74–0.86)	0.76	(0.45–0.74)	0.58*	(0.38–0.88)
Q5 (Most)	0.80***	(0.74–0.87)	0.70	(0.39–0.69)	0.55*	(0.35–0.87)
Total Observations (N)	*743,746*		*1,243,932*		*1,243,932*	

(Continued)

Supplemental Material 7 | *(Continued).*

	PHC usage		Hospitalisation		Mortality	
**Characteristics**	**ARR**	**95% CI**	**ARR**	**95% CI**	**ARR**	**95% CI**
INTERACTIONS						
**Race/Colour × Deciles of Income**						
Black ×Q2	1.03	(0.76–1.39)	0.83	(0.35–1.94)	0.90	(0.22–3.59)
Black × Q3	1.07	(0.78–1.47)	0.52	(0.23–1.20)	1.24	(0.32–4.80)
Black × Q4	1.12	(0.83–1.51)	0.97	(0.41–2.32)	0.79	(0.17–3.65)
Black × Q5	0.92	(0.68–1.24)	1.07	(0.45–2.53)	1.56	(0.34–7.14)
Black × Q6	1.13	(0.84–1.52)	0.51	(0.22–1.20)	3.08	(0.51–18.76)
Black × Q7	1.27	(0.92–1.76)	0.35*	(0.14–0.85)	0.35	(0.05–2.31)
Black × Q8	0.95	(0.70–1.29)	0.44	(0.18–1.07)	1.20	(0.28–5.13)
Black × Q9	1.10	(0.81–1.48)	0.61	(0.27–1.42)	0.27	(0.04–1.68)
Black × Q10	1.18	(0.90–1.54)	0.30**	(0.14–0.61)	0.49	(0.13–1.83)
Pardo (Mixed) × Q2	1.08	(0.86–1.36)	1.02	(0.53–1.96)	0.73	(0.21–2.47)
Pardo (Mixed) × Q3	1.19	(0.95–1.49)	1.03	(0.52–2.05)	0.53	(0.15–1.88)
Pardo (Mixed) × Q4	1.06	(0.84–1.33)	0.81	(0.42–1.58)	0.65	(0.17–2.42)
Pardo (Mixed) × Q5	0.96	(0.76–1.21)	0.92	(0.48–1.76)	0.79	(0.20–3.21)
Pardo (Mixed) × Q6	0.95	(0.76–1.19)	0.51	(0.23–1.13)	1.11	(0.19–6.40)
Pardo (Mixed) × Q7	0.93	(0.74–1.16)	0.35**	(0.16–0.76)	0.59	(0.15–2.27)
Pardo (Mixed) × Q8	1.19	(0.95–1.48)	0.85	(0.41–1.78)	0.64	(0.17–2.41)
Pardo (Mixed) × Q9	0.98	(0.80–1.21)	0.87	(0.45–1.69)	0.35	(0.10–1.31)
Pardo (Mixed) × Q10	1.22*	(1.00–1.50)	0.63	(0.35–1.16)	0.61	(0.21–1.75)
Other × Q2	1.97	(0.77–5.06)	0.83	(0.18–3.86)	0.00***	(0.00–0.00)
Other × Q3	1.46	(0.61–3.48)	0.51	(0.09–3.00)	0.00***	(0.00–0.00)
Other × Q4	1.58	(0.76–3.31)	1.21	(0.31–4.79)	3.79	(0.19–76.11)
Other × Q5	1.17	(0.62–2.22)	1.05	(0.21–5.34)	0.00***	(0.00–0.00)
Other × Q6	1.39	(0.66–2.94)	0.40	(0.08–1.89)	0.00***	(0.00–0.00)
Other × Q7	1.79	(0.89–3.61)	0.48	(0.11–2.23)	0.00***	(0.00–0.00)
Other × Q8	1.58	(0.75–3.30)	0.13*	(0.02–0.77)	3.61	(0.18–72.36)
Other × Q9	1.57	(0.88–2.78)	1.35	(0.24–7.72)	0.00***	(0.00–0.00)
Other × Q10	2.39**	(1.40–4.09)	0.53	(0.16–1.72)	0.82	(0.04–15.55)
Total Observations (N)	*743,746*		*1,243,932*		*1,243,932*	
	**PHC usage**		**Hospitalisation**		**Mortality**	
Overall Interaction Significance	*		*		***	

(Continued)

Supplemental Material 7 | (*Continued).*

	PHC usage		Hospitalisation		Mortality	
**Characteristics**	**ARR**	**95% CI**	**ARR**	**95% CI**	**ARR**	**95% CI**
**Race/Colour × Deciles of Income** *Fully Expanded*						
White × Q1	1 (ref)	–	1 (ref)	–	1 (ref)	–
White × Q2	0.90	(0.76–1.08)	0.83	(0.53–1.30)	0.95	(0.53–1.30)
White × Q3	0.82*	(0.69–0.97)	0.95	(0.55–1.61)	0.87	(0.55–1.61)
White × Q4	0.93	(0.78–1.10)	0.81	(0.50–1.33)	0.72	(0.50–1.33)
White × Q5	0.92	(0.77–1.10)	0.68	(0.42–1.12)	0.53	(0.42–1.12)
White × Q6	0.84*	(0.71–1.00)	1.18	(0.69–2.01)	0.27	(0.69–2.01)
White × Q7	0.90	(0.75–1.07)	1.91*	(1.02–3.56)	0.66	(1.02–3.56)
White × Q8	0.81*	(0.68–0.96)	1.36	(0.74–2.50)	0.64	(0.74–2.50)
White × Q9	0.93	(0.79–1.09)	1.26	(0.73–2.18)	0.76	(0.73–2.18)
White × Q10	0.94	(0.79–1.10)	2.98***	(1.79–4.95)	0.91	(1.79–4.95)
Black × Q1	0.59***	(0.47–0.73)	1.73	(0.99–3.03)	1.90	(0.99–3.03)
Black × Q2	0.55***	(0.44–0.67)	1.18	(0.62–2.24)	1.62	(0.62–2.24)
Black × Q3	0.51***	(0.40–0.65)	0.86	(0.50–1.45)	2.05	(0.50–1.45)
Black × Q4	0.61***	(0.50–0.75)	1.36	(0.73–2.54)	1.08	(0.73–2.54)
Black × Q5	0.49***	(0.40–0.61)	1.26	(0.68–2.33)	1.57	(0.68–2.33)
Black × Q6	0.56***	(0.46–0.69)	1.04	(0.58–1.85)	1.57	(0.58–1.85)
Black × Q7	0.67**	(0.53–0.85)	1.15	(0.66–2.00)	0.44	(0.66–2.00)
Black × Q8	0.45***	(0.36–0.56)	1.03	(0.56–1.89)	1.45	(0.56–1.89)
Black × Q9	0.60***	(0.48–0.74)	1.34	(0.75–2.39)	0.40	(0.75–2.39)
Black × Q10	0.64***	(0.53–0.79)	1.52	(0.91–2.52)	0.85	(0.91–2.52)
Pardo (Mixed) × Q1	0.82*	(0.70–0.96)	1.14	(0.72–1.80)	1.91	(0.72–1.80)
Pardo (Mixed) × Q2	0.80**	(0.68–0.93)	0.96	(0.61–1.51)	1.32	(0.61–1.51)
Pardo (Mixed) × Q3	0.79**	(0.68–0.93)	1.11	(0.73–1.68)	0.88	(0.73–1.68)
Pardo (Mixed) × Q4	0.80***	(0.67–0.96)	0.75	(0.49–1.15)	0.89	(0.49–1.15)
Pardo (Mixed) × Q5	0.72***	(0.61–0.85)	0.72	(0.48–1.07)	0.80	(0.48–1.07)
Pardo (Mixed) × Q6	0.66***	(0.56–0.77)	0.68	(0.38–1.22)	0.57	(0.38–1.22)
Pardo (Mixed) × Q7	0.68***	(0.58–0.80)	0.77	(0.49–1.20)	0.74	(0.49–1.20)
Pardo (Mixed) × Q8	0.78**	(0.66–0.92)	1.32	(0.84–2.06)	0.78	(0.84–2.06)
Pardo (Mixed) × Q9	0.74***	(0.63–0.87)	1.25	(0.82–1.89)	0.51	(0.82–1.89)
Pardo (Mixed) × Q10	0.93	(0.79–1.10)	2.15**	(1.34–3.42)	1.06	(1.34–3.42)
Other × Q1	0.49**	(0.31–0.79)	0.82	(0.30–2.23)	0.87	(0.30–2.23)
Other × Q2	0.88	(0.39–1.99)	0.57	(0.17–1.84)	0.00***	(0.17–1.84)
Other × Q3	0.59	(0.28–1.22)	0.39	(0.09–1.69)	0.00***	(0.09–1.69)
Other × Q4	0.72	(0.41–1.28)	0.81	(0.32–2.08)	2.39	(0.32–2.08)
Other × Q5	0.53**	(0.35–0.81)	0.59	(0.16–2.16)	0.00***	(0.16–2.16)
Other × Q6	0.58	(0.32–1.03)	0.38	(0.12–1.26)	0.00***	(0.12–1.26)
Other × Q7	0.79	(0.47–1.32)	0.76	(0.25–2.28)	0.00***	(0.25–2.28)
Other × Q8	0.63	(0.35–1.11)	0.15**	(0.04–0.62)	2.03	(0.04–0.62)
Other × Q9	0.71*	(0.51–1.00)	1.40	(0.34–5.73)	0.00***	(0.34–5.73)
Other × Q10	1.10	(0.83–1.45)	1.30	(0.65–2.60)	0.66	(0.65–2.60)
Total Observations (N)	*743,746*		*1,243,932*		*1,243,932*	

PHC – Primary Healthcare; ARR – Adjusted Rate Ratios; 95% CI – 95% Confidence Intervals.
Separate fully adjusted Poisson regressions per outcome (PHC usage [ESF registered users only], hospitalisation, and mortality); adjusted for sex, education level, age group, disability, unemployment, number of family members per bedroom, household flooring, household piped water access, formal employment in the family, Bolsa Família-receiving family, quintiles of household expenditure on medicines and food.
*Robust standard errors. *p<0.05; **p<0.01; *** p<0.001.*
