# Supplementary material for: Racial inequalities in mental healthcare use and mortality: a cross-sectional analysis of 1.2 million low-income individuals in Rio de Janeiro, Brazil 2010–2016
Source: BMJ Glob Health. 2023 Dec 2;8(12):e013327. doi: 10.1136/bmjgh-2023-013327 (PMC10693873; doi:10.1136/bmjgh-2023-013327)
Supplement: Supplementary data [file bmjgh-2023-013327supp008.pdf]

Supplemental Material 8 | Predicted rates per 100,000 person-years based on adjusted Poisson regression models for interactions between race/colour and deciles of income.

Characteristics	PHC usage		Hospitalisation		Mortality	
	Predicted Rate (per 100,000 person-years)	95% CI	Predicted Rate (per 100,000 person-years)	95% CI	Predicted Rate (per 100,000 person-years)	95% CI
**Race/Colour × Deciles of Income**						
White × Q1	7,118.4***	(6,227.21–8,009.51)	129.1***	(87.51–170.64)	3.8**	(1.15–6.52)
White × Q2	6,441.5***	(5,583.66–7,299.43)	106.8***	(70.59–143.10)	3.6*	(0.72–6.57)
White × Q3	5,815.2***	(5,080.30–6,550.15)	122.1***	(69.23–174.87)	3.3*	(0.63–6.06)
White × Q4	6,611.5***	(5,820.74–7,402.27)	105.0***	(64.78–145.31)	2.8*	(0.32–5.20)
White × Q5	6,552.2***	(5,719.74–7,384.69)	88.2***	(54.16–122.27)	2.0*	(0.03–4.02)
White × Q6	5,995.2***	(5,281.45–6,708.93)	152.1***	(86.96–217.30)	1.0	(-0.40–2.47)
White × Q7	6,377.8***	(5,594.92–7,160.63)	246.0***	(114.81–377.19)	2.5*	(0.29–4.75)
White × Q8	5,743.0***	(5,081.74–6,404.30)	176.1***	(88.03–264.14)	2.5*	(0.30–4.62)
White × Q9	6,596.3***	(5,916.44–7,276.16)	162.3***	(93.50–231.05)	2.9**	(0.74–5.10)
White × Q10	6657.6***	(6,021.42–7,293.83)	384.2***	(251.58–516.82)	3.5**	(1.45–5.57)
Black × Q1	4172.0***	(3,405.53–4,938.43)	223.0***	(116.67–329.37)	7.3**	(3.02–11.52)
Black × Q2	3,886.4***	(3,225.91–4,546.91)	152.4**	(65.29–239.49)	6.2**	(1.76–10.65)
Black × Q3	3,642.3***	(2,898.85–4,385.71)	110.6***	(62.94–158.28)	7.9**	(2.95–12.76)
Black × Q4	4,348.0***	(3,598.17–5,097.89)	176.1***	(79.41–272.79)	4.1*	(0.49–7.76)
Black × Q5	3,519.0***	(2,923.54–4,114.39)	162.3***	(75.43–249.23)	6.0**	(1.49–10.52)
Black × Q6	3,980.9***	(3,337.03–4,624.84)	133.8***	(68.04–199.54)	6.0**	(1.50–10.57)
Black × Q7	4,764.4***	(3,801.45–5,727.43)	147.9***	(80.40–215.40)	1.7	(-0.67–4.06)
Black × Q8	3,200.1***	(2,626.31–3,773.91)	132.6***	(64.37–200.76)	5.6**	(1.45–9.69)
Black × Q9	4,244.4***	(3,489.66–4,999.05)	172.4***	(91.16–253.62)	1.5	(-0.60–3.64)
Black × Q10	4,588.7***	(3,917.26–5,260.20)	195.9***	(123.48–268.26)	3.3*	(0.59–5.94)
Pardo (Mixed) × Q1	5,807.2***	(5,189.79–6,424.59)	146.6***	(93.73–199.50)	7.3***	(4.53–10.12)
Pardo (Mixed) × Q2	5,663.5***	(5,045.81–6,281.25)	123.8***	(81.32–166.19)	5.1***	(2.47–7.68)
Pardo (Mixed) × Q3	5,636.9***	(5,047.03–6,226.76)	143.2***	(103.02–183.30)	3.4**	(1.36–5.43)
Pardo (Mixed) × Q4	5,697.3***	(4,976.52–6,418.10)	97.1***	(68.70–125.57)	3.4**	(1.37–5.49)
Pardo (Mixed) × Q5	5,139.6***	(4,579.49–5,699.76)	92.6***	(68.82–116.34)	3.1**	(1.11–5.03)
Pardo (Mixed) × Q6	4,669.2***	(4,174.41–5,163.94)	87.8***	(43.75–131.90)	2.2**	(0.57–3.80)
Pardo (Mixed) × Q7	4,842.2***	(4,352.42–5,332.00)	98.8***	(67.43–130.09)	2.8**	(0.99–4.69)
Pardo (Mixed) × Q8	5,555.6***	(4,942.67–6,168.59)	170.1***	(117.47–222.82)	3.0**	(1.12–4.88)
Pardo (Mixed) × Q9	5,274.8***	(4,815.80–5,733.83)	161.1***	(122.43–199.81)	2.0**	(0.50–3.45)
Pardo (Mixed) × Q10	6,651.6***	(6,007.89–7,295.37)	276.9***	(194.94–358.84)	4.1***	(1.96–6.15)
Other × Q1	3,498.6***	(1,886.65–5,110.49)	106.2*	(5.70–206.67)	3.4	(-3.41–10.12)
Other × Q2	6,235.3*	(1,172.80–11,297.89)	73.3	(-9.87–156.42)	0.0***	(0.00–0.00)
Other × Q3	4,175.6**	(1,161.14–7,190.12)	50.9	(-21.50–123.34)	0.0***	(0.00–0.00)
Other × Q4	5,144.6***	(2,270.04–8,019.25)	104.8*	(11.70–197.91)	9.2	(-8.77–27.07)
Other × Q5	3,775.3***	(2,223.62–5,327.04)	76.2	(-19.27–171.69)	0.0***	(0.00–0.00)
Other × Q6	4,103.1**	(1,753.38–6,452.85)	49.6	(-6.94–106.09)	0.0***	(0.00–0.00)
Other × Q7	5,624.3***	(2,799.56–8,448.97)	98.1	(-4.82–201.00)	0.0***	(0.00–0.00)
Other × Q8	4,453.8***	(1,970.17–6,937.42)	19.1	(-7.47–45.72)	7.8	(-7.53–23.06)
Other × Q9	5,074.1***	(3,513.00–6,635.17)	180.7	(-66.36–427.75)	0.0***	(0.00–0.00)
Other × Q10	7,821.6***	(5,907.81–9,735.39)	168.1**	(69.01–267.23)	2.5	(-2.42–7.47)

PHC – Primary Healthcare; 95% CI – 95% Confidence Intervals.
*Obtained from separate fully adjusted Poisson regressions per outcome (PHC usage [registered users only], hospitalisation, and mortality); adjusted for sex, education level, age group, disability, unemployment, number of family members per bedroom, household flooring, household piped water access, formal employment in the family, Bolsa Família-receiving family, quintiles of household expenditure on medicines and food. *p<0.05; **p<0.01; *** p<0.001.*
