# Supplementary material for: Racial inequalities in mental healthcare use and mortality: a cross-sectional analysis of 1.2 million low-income individuals in Rio de Janeiro, Brazil 2010–2016
Source: BMJ Glob Health. 2023 Dec 2;8(12):e013327. doi: 10.1136/bmjgh-2023-013327 (PMC10693873; doi:10.1136/bmjgh-2023-013327)
Supplement: Supplementary data [file bmjgh-2023-013327supp010.pdf]

Reflexivity Statement

1. **How does this study address local research and policy priorities?**

This study was designed to address racial and ethnic health inequalities in mental health. Poor mental health is a significant problem in Brazil, yet there is a dearth of evidence on the depth and complexities of racial and ethnic inequalities in mental health outcomes from the global south – a pressing evidence gap for driving policy change. Mental disorders are the third cause of disease burden in Brazil, behind only cardiovascular diseases and cancer. Few studies have explored associations between race and mental health care utilisation and mental health outcomes in Brazil, with previous work producing mixed findings. Rio de Janeiro is an important setting for this research. It is highly unequal city with a GINI index of 0.6392 in 2010. Our study examines the individual-level health records of 1.2 million low-income individuals in Rio de Janeiro, Brazil over a seven-year period to document racial inequalities in mental healthcare use and mental health outcomes - a major novelty for a LMIC. It addresses an evidence gap around two pressing global challenges – mental health and racial/ethnic inequalities – using rich individual-level data to not only quantify these inequalities but demonstrate the power of investments in high-quality data systems for health.

2. **How were local researchers involved in study design?**

All local researchers were involved in conceptualisation of this study, either through curation of the data or conceptual thinking using local knowledge. Brazilian authors obtained the data and built the datasets, establishing a legacy dataset for ongoing studies, as well as having contributed to prior studies using the data.

3. **How has funding been used to support the local research team?**

Funding for the construction of this dataset was used to support local research teams through salaries and infrastructure costs.

4. **How are research staff who conducted data collection acknowledged?**

This study utilises only secondary data. All researchers involved in the data curation were included as authors.

5. **Do all members of the research partnership have access to study data?**

All authors, with the exception of RC have access to the data.

6. **How was data used to develop analytical skills within the partnership?**

All co-authors have been involved in the analyses that underpin this study and other related studies using this dataset. SM, the early career researcher and first author, conducted the formal data analysis, allowing them to develop their analytical skills.

7. How have research partners collaborated in interpreting study data?

All researchers reviewed and provided critical interpretation of the results.

8. How were research partners supported to develop writing skills?

The early career researcher (SM) wrote the original draft. All co-authors contributed to editing and reviewing of manuscript.

9. How will research products be shared to address local needs?

A local language abstract is provided in the supplemental material. The paper will be shared directly with journalists in Brazil and through civil society organisations to reach local audiences.

10. How is the leadership, contribution and ownership of this work by LMIC researchers recognised within the authorship?

All Brazilian authors were involved in data curation, project leadership, and involved as co-authors.

11. How have early career researchers across the partnership been included within the authorship team?

The first author is an early career researcher.

12. How has gender balance been addressed within the authorship?

Five authors are female (SM, VS, CMC, AT, BD) and four are male (RC, CM, DR, TH).

13. How has the project contributed to training of LMIC researchers?

The authorship team is composed of researchers across all career levels. Authors from LMICs are primarily mid-career or senior researchers.

14. How has the project contributed to improvements in local infrastructure?

The funding for this study contributed to investments in IT infrastructure at the Federal University of Rio de Janeiro.

15. What safeguarding procedures were used to protect local study participants and researchers?

This study did not involve primary data collection, however all datasets were anonymised after linkage and held securely by local researchers.
